# Optimal Grazing Exclusion Duration to Enhance Soil Carbon Sequestration in Degraded Grasslands

**DOI:** 10.1002/advs.202522212

**Published:** 2026-03-12

**Authors:** Bin Zhang, Chongzhi Sun, Dongmei Xue, Paul Christiaan Struik, Tongrui Zhang, Jiayue Liu, Tingting Xing, Ziyang Liu, Shiming Tang, Ke Jin

**Affiliations:** ^1^ Key Laboratory for Model Innovation in Forage Production Efficiency Ministry of Agriculture and Rural Affairs Institute of Grassland Research Chinese Academy of Agricultural Sciences Hohhot China; ^2^ Department of Grassland Science College of Grassland Science and Technology China Agricultural University Beijing China; ^3^ Tianjin Key Laboratory of Water Resources and Environment Tianjin Normal University Tianjin China; ^4^ Centre for Crop Systems Analysis Department of Plant Sciences Wageningen University & Research Wageningen Netherlands

**Keywords:** fencing, grassland restoration, grazing exclusion duration, precipitation dependence, soil carbon content

## Abstract

Soil organic carbon (SOC) plays a crucial role in mitigating climate change and ensuring food security. Grazing exclusion (GE) is widely recognized as an effective strategy for restoring degraded grasslands and enhancing SOC sequestration, yet the optimal duration for this practice remains uncertain. Here, we conducted a meta‐analysis using 4257 paired observations from 125 studies in China to quantify the impact of GE on SOC content and stock, and to determine the optimal duration for maximizing carbon sequestration. We found that GE increased SOC content and stock by an average of 34.12% and 24.56%, respectively. This positive effect of GE on SOC becomes greater over time in areas with higher mean annual precipitation (MAP), but it is negative below 200 mm. These effects of GE remain consistent across alpine, meadow, desert, and typical grasslands, and are independent of other environmental attributes. Our results indicate that implementing GE on 70% of degraded grasslands nationwide could, on average, sequester 1.52 Pg C in soil over a period of 10 years (about 17% of global annual fossil fuel emissions), with larger gains expected in higher‐MAP regions. Our synthesis provides an evidence‐based framework for optimizing grassland restoration through enhanced soil carbon sequestration in degraded grasslands.

## Graphical abstract

Across China, grazing exclusion reaches the national mean soil organic carbon recovery benchmark sooner in high‐MAP regions (> 500 mm), but recovery is much slower where MAP < 300 mm. Scaling this strategy to 70% of China's degraded grasslands would sequester about 1.52 Pg of soil carbon over 10 years—roughly 17% of annual global fossil‐fuel emissions.

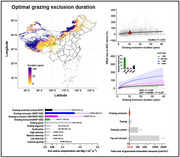

## 1 Introduction

Soil organic carbon (SOC), as a crucially important natural resource, provides essential ecosystem services, including climate change mitigation and food security assurance [1]. The global SOC stock is roughly three times the combined carbon content of the atmosphere and vegetation, and even minor changes in its dynamics can have profound impacts on the global carbon cycle [2]. Grasslands store approximately 34% of the terrestrial carbon stock [3], but extensive human utilization and inadequate protection have led to degradation in 49% of the global grasslands [4], with 70% in China's grasslands affected [5]. The severe grassland degradation has substantially reduced the soil carbon sequestration potential of grasslands, leading to significant loss of soil carbon [6]. In this context, nature‐based solutions are particularly important for addressing long‐term challenges, especially in grassland restoration [7]. Identifying effective, cost‐efficient, and scalable restoration measures is therefore crucial for restoring grassland carbon stocks.

As a typical nature‐based solution, grazing exclusion (GE) is widely adopted for SOC restoration in degraded grasslands [8]. However, most studies have only confirmed the positive effects of GE on soil carbon sequestration, accurately quantifying the optimal GE duration remains unclear [9, 10]. An overly short‐term GE may fail to achieve the desired restoration, while an excessively long duration can lead to resource wastage due to the delayed utilization post‐SOC equilibrium. Plants play a decisive role in the recovery of SOC, balancing the soil carbon input and output [11]. After GE, the elimination of herbivore grazing retains plant aboveground biomass (AGB), increasing carbon assimilation and allocation to roots, thus enhancing SOC [12]. In addition, reduced trampling under GE can decrease soil bulk density, thereby facilitating root growth and microbial activity [13]. The increased litter and root input boost SOC in the early stage of GE [14]. However, prolonged GE results in a thick litter layer, reducing photodegradation and intensifying plant competition for light and nutrients, leading to community homogenization and stable biomass [15]. Besides, continuous litter input may increase the priming effect on decomposition of SOC by stimulating microbial activities, and thus accelerating soil CO_2_ effluxes and slowing SOC increase [16].

The fundamental value of grassland lies in its utilization, so the duration of GE should not be unduly prolonged. Defining this duration for SOC to reach an optimum restoration effect is crucial for the efficient utilization of resources and the best possible carbon sequestration. Yet, robust quantitative studies in this area are limited, especially of the impact of precipitation. In arid and semi‐arid grasslands, water availability represents a dominant climatic constraint on both carbon inputs and turnover; therefore, mean annual precipitation provides a practical and widely harmonizable proxy to characterize climatic modulation of GE‐induced SOC recovery, alongside other important modifiers such as soil depth, MAT, and grassland type. Although water availability is a well‐established regulator of both plant productivity and microbial decomposition, the role of precipitation in modulating the effectiveness of GE on SOC remains largely underexplored [17, 18]. In arid and semi‐arid grasslands, plant growth and belowground carbon inputs are strongly constrained by water availability. Moreover, drought or wet conditions further influence microbial activity by altering resource diffusion, transport, and organismal stress responses [19, 20]. These cascading effects alter key biogeochemical processes, ultimately impacting the decomposition of plant litter and existing soil organic matter. As a dominant climatic driver of water availability, mean annual precipitation (MAP) plays a central role in modulating SOC dynamics under GE [21]. Notably, GE duration and MAP jointly influence the balance between carbon inputs from plant litter and root exudates and carbon losses via microbial mineralization [22]. In high‐MAP regions, favorable water conditions accelerate SOC recovery following GE, allowing degraded grasslands to reach stable carbon levels more rapidly. Conversely, low‐MAP regions experience delayed or even stagnant SOC restoration, potentially requiring much longer GE duration to achieve comparable outcomes [9, 23, 24]. Despite these dynamics, the interactive effects of MAP and GE duration on SOC accumulation remain poorly quantified, limiting the development of climate‐specific GE strategies.

The regulatory effect of GE on the SOC restoration may also change with the soil depth, the mean annual temperature (MAT), and grassland types. Due to soil organic matter availability, microbial community structure, and soil properties differing significantly with soil depth, the factors that mediate soil organic matter composition at least partly differ in the topsoil and subsoil, which may lead to the effect of GE on SOC varying with soil depth [12, 25]. The fluctuations of the MAT are capable of modifying the evaporation rate of the soil surface, the photosynthetic efficiency of vegetation, and the activity of soil microorganisms. It may either weaken or intensify the influence of GE on SOC by regulating plant growth, decomposition rates, and soil respiration rates [26, 27]. Differences in the dominant microbes between different grassland types, which are related to microbial preferences for hydrothermal conditions, habitat, soil environment, nutrient conditions, and vegetation community composition, suggest that the effect of GE on SOC may vary among grassland types [28, 29].

Here, we conducted a comprehensive meta‐analysis of 4257 paired observations from 125 studies across China's grassland ecosystems (Figure S1 and Table S1). Notably, we hypothesize that GE duration and MAP jointly influence the balance between carbon inputs from plant litter and root exudates and carbon losses via microbial mineralization. Accordingly, we expect that in high‐MAP regions, favorable water conditions can facilitate plant inputs and microbial processing, potentially allowing faster SOC recovery following GE, whereas in low‐MAP regions, SOC restoration may be delayed or even constrained, implying that longer GE duration may be required to achieve comparable outcomes. Our study has two primary objectives: (1) to quantify GE‐driven SOC dynamics across China's grassland ecosystems; (2) to test whether and quantify how precipitation modulates the optimal GE duration for SOC sequestration. By integrating these analyses, we provide the first evidence‐based guidelines for optimizing GE duration according to local precipitation regimes – a crucial advancement for grassland carbon management. Our findings not only refine the ecological theory on SOC stabilization but also offer immediate policy relevance. With China's GE programs already covering 26.2 million hectares, our results can directly inform the scaling of restoration efforts nationally, while the underlying principles apply globally to grassland ecosystems facing similar degradation and climate change pressures.

## 2 Results

### 2.1 The Average Effects of Grazing Exclusion on Environmental Attributes

**FIGURE 1:**
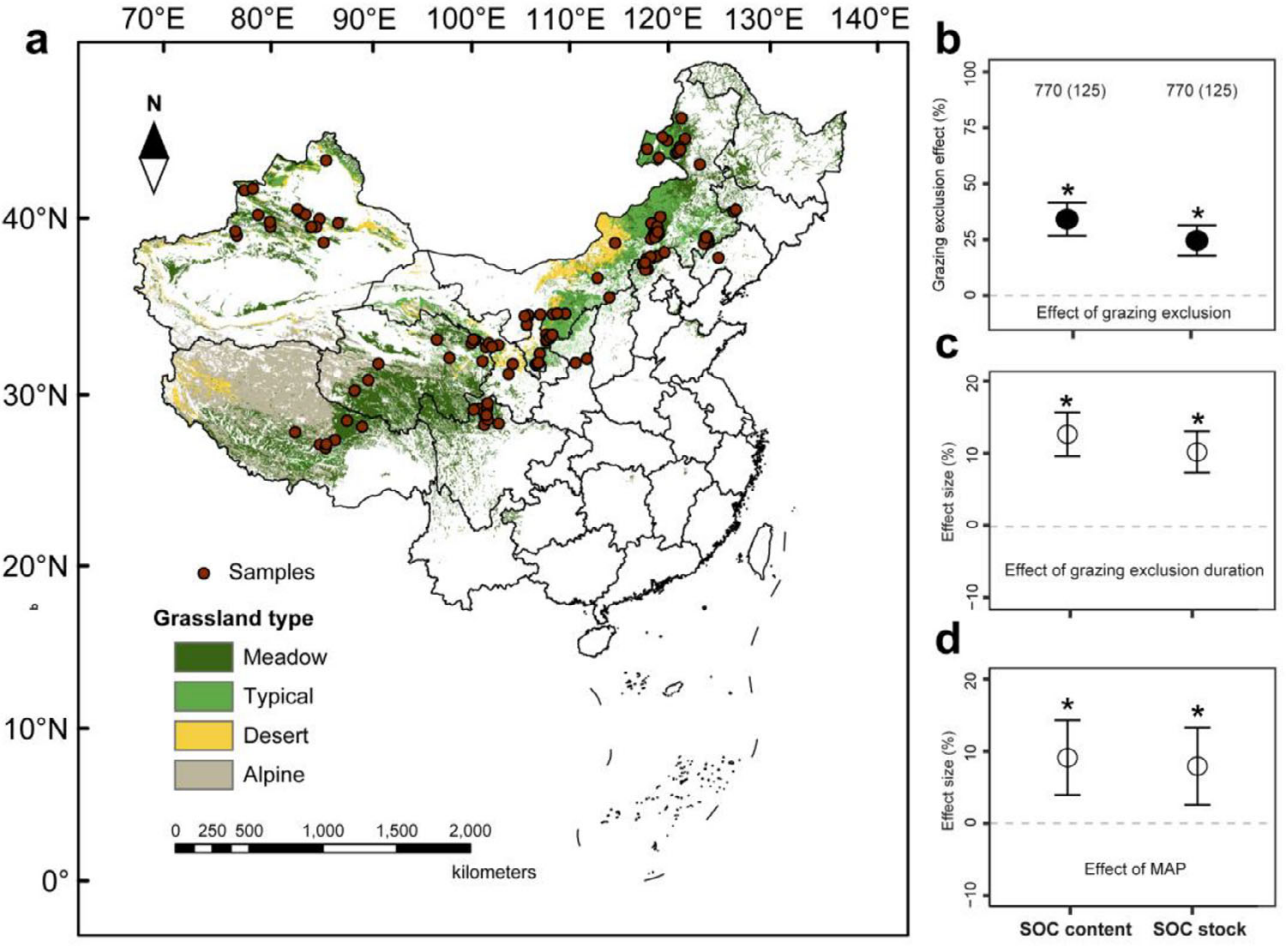
The network of experiments to investigate the impacts of grazing exclusion on soil organic carbon (SOC) in China. (a) Distribution of study sites in the meta‐analysis. (b) Comparison of SOC attributes for grazing exclusion versus grazing. (c) In relation to grazing exclusion duration. (d) In relation to mean annual precipitation (MAP). The grazing exclusion effects in (b) represent the increase or decrease (%) of a given SOC attribute compared to the corresponding mean of grazing at the average durations and precipitation. The grazing exclusion duration effects in (c) and the mean annual precipitation effects in (d) represent the estimated coefficients of the grazing exclusion in duration and precipitation, respectively. The number of observations in (b) is shown without parentheses above each attribute, together with the number of studies in parentheses. Values in b, c, and d are means ± 95% confidence intervals. Asterisks indicate significant difference or effect size at *P* < 0.05. Source data are provided as a Source Data file.

The GE effect on environmental attributes did not differ significantly among grassland types, including alpine, typical, desert, and meadow (Figure S2), except for bulk density. Across all four grassland types, SOC content increased significantly, on average by 34.12% (95% confidence interval, CI, 26.73 – 41.52%), while the SOC stock increased by 24.56% (CI 17.79 – 31.33%), AGB by 100.31% (CI 17.93 – 182.69%), belowground biomass (BGB) by 89.01% (CI 3.44 – 174.58%), total nitrogen by 28.85% (CI 20.83 – 36.86%), and total phosphorus by 31.45% (CI 19.88 – 43.03%), but soil pH decreased significantly on average by 2.07% (CI ‐3.18 – ‐0.95%) and bulk density by 7.61% (CI ‐8.61 – ‐6.61%) in grazing exclusion compared with the mean of grazing (Figure 1b and Figure S3a).

### 2.2 The Variation of Mean Annual Precipitation Effects

With increasing MAP, the effect sizes for SOC content (CI 3.72–14.44%), SOC stock (CI 2.76–13.12%), and total phosphorus (CI 1.69–16.14%) significantly increased, but the effect size for bulk density (CI ‐1.96 – ‐13.41%) decreased with the precipitation levels, while the AGB, BGB, total nitrogen and soil pH showed no significant responses to MAP (Figure 1b and Figure S3b).

### 2.3 The Variation of Grazing Exclusion Duration Effects

With longer grazing exclusion duration, the effect sizes for SOC content (CI 9.77 – 15.84%), SOC stock (CI 7.48 – 13.22%), total nitrogen (CI 6.63 – 13.85%), and total phosphorus (CI 1.77 – 8.76%) significantly increased, but the effect size of soil pH (CI ‐2.29 – ‐1.13%) and bulk density (CI ‐2.53 – ‐1.60%) decreased with the GE duration, and the plant biomass showed no significant responses to GE duration (Figure 1c and Figure S3c).

### 2.4 Relationship Between Grazing Exclusion Duration and Mean Annual Precipitation in SOC Content and SOC Stock

**FIGURE 2:**
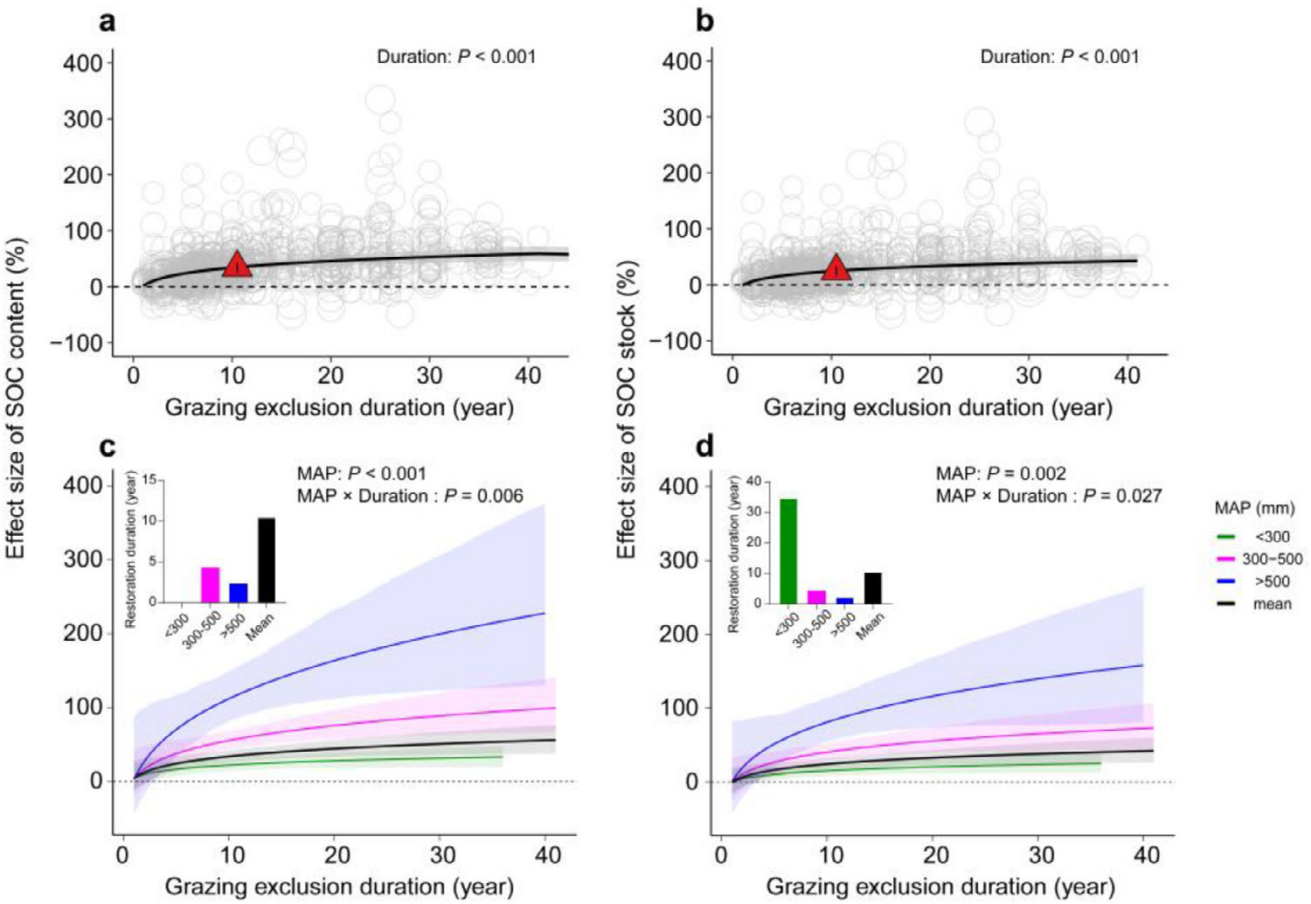
Comparison of soil organic carbon content and stock under grazing versus grazing exclusion. (**a** and **b)** represent the soil organic carbon content and stock in relation to grazing exclusion duration. (**c** and **d)** represent the interactive effects of the grazing exclusion duration and mean annual precipitation (MAP) on soil organic carbon content and stock, respectively. The effects are quantified as the percentage changes under grazing exclusion compared with the corresponding mean value under grazing. Slope estimates in (**a** and **b)** are partial dependence, derived from the full model (see Methods), and red triangles and error bars represent the overall means and their 95% confidence intervals, respectively. Circles represent the values predicted by partial regressions for each explanatory variable, with their sizes representing the relative weights of corresponding observations. Black and colored lines in (**c** and **d)** represent the average and MAP‐specific responses, respectively, with their bootstrapped 95% confidence intervals indicated by shading. Green, purple, and blue lines in (**c** and **d)** represent the mean annual precipitation for regions receiving less than 300 mm, between 300 and 500 mm, and more than 500 mm, respectively. The black line indicates the overall mean annual precipitation across all observed regions.

As indicated in Table S2, the responses of SOC content, SOC stock, total nitrogen, total phosphorus, and pH to GE were generally consistent across soil depth, mean annual temperature (MAT), and grassland type (alpine, typical, desert, and meadow). This consistency applies to both the average responses and the estimated effects of GE duration and MAP. Therefore, we present these results pooled across all observations unless stated otherwise. By contrast, AGB and BGB were influenced by the interaction between grassland type and GE duration (Table S2). On average across all sites, GE increased SOC content and SOC stock by 34.45% (CI 25.94–42.95%) and 24.60% (CI 17.41–31.80%), respectively (Figure 2). Linear regression analysis showed that AGB, BGB, soil total nitrogen, total phosphorus, microbial biomass carbon and β‐glucosidase were significantly positively correlated with SOC, while soil bulk density, microbial biomass nitrogen and peroxidase were significantly negatively correlated with SOC (Figure S7). The GE effects on SOC content and SOC stock were positive since the beginning of the GE, and the average level of the restored SOC was reached approximately 10 years after establishing the fencing, similar among different regions varying in MAP levels. The GE effect on SOC content and SOC stock reached average restoration over time, with an average effect of 34.19% (CI 26.32 – 42.06%) and 24.79% (CI 17.37 – 32.20%) (Figure 2a, b).

Both SOC content and SOC stock increased with the extension of GE duration, with higher MAP levels amplifying this trend. Only regions with MAP < 300 mm consistently showed recovery effects below the average (Figure 2a, b). Model results indicate that in regions with MAP < 300 mm, SOC content would not reach the average recovery level, even not after 42 years of GE. In contrast, regions with MAP between 300 and 500 mm only required 4.35 years of GE, and those with MAP > 500 mm only needed 2.35 years of GE to reach the average SOC content. Similarly, restoring SOC stock in regions with MAP < 300 mm would take 34.74 years, while regions with MAP between 300 and 500 mm and MAP > 500 mm would need 4.49 and 2.35 years, respectively (Figure 2a, b). Higher MAP not only accelerated SOC recovery but also significantly reduced the duration required for GE to reach its average carbon sequestration potential. In regions with MAP > 500 mm, this reduction exceeded 93.23% compared to MAP < 300 mm (Figure 2a, b). In addition, we found that in areas with MAP < 193 mm and 205 mm, GE measures could not improve SOC content and stock at the average years of GE duration (Figure S9).

The map illustrates optimal GE durations, calculated using our fitted model relating GE duration to MAP. Optimal durations represent the minimal number of years required to achieve average SOC recovery effects. MAP values used for predictions were derived from the average of annual precipitation data from 2000 to 2023. The analysis identifies substantial regional differences in GE requirements, with shorter durations (< 10 years) in humid regions of eastern and southern China, intermediate durations (10–25 years) in semi‐arid central regions, and significantly prolonged durations (> 50 years) predominantly in the north of Inner Mongolia, Tibet, and southern Xinjiang. These spatially explicit required durations provide a data‐driven basis for tailoring GE strategies to local precipitation conditions, enhancing the efficiency and effectiveness of grassland restoration and carbon sequestration initiatives nationwide

### 2.5 Predicted Responses of SOC Stock

Based on the fitted responses of precipitation and GE duration (Figure 4), a decrease in MAP by 10%, 20%, 40%, and an extreme 80% (from 100% to 90%, 80%, 60%, and 20%) over one year led to reductions in GE's effect on SOC stock by 1.13% to 15.97%. These reductions progressively increased over five years to 2.49%–32.01%, and after ten years, reached 4.17%–47.82%. The decline in SOC stock recovery became more pronounced with extended GE duration, especially under lower MAP (Figure 4a). However, under extreme MAP reductions, the curve flattened around 40 years. Conversely, increases in MAP by the same percentages enhanced the GE effect on SOC stock. Over one year, the enhancement ranged from 1.04% to 6.56%, increasing to 2.31%–15.13% over five years, and reaching 3.93%–26.81% after ten years (Figure 4b). The positive impact of GE on SOC stock accelerated with both higher MAP and longer GE durations. These results indicate that while both increases and decreases in MAP significantly affect SOC stock recovery by GE, the negative effect of extreme reductions in MAP plateaus as the GE duration extends.

### 2.6 Horizontal Comparison of Management on Soil Carbon Sequestration Rate

The soil carbon sequestration rate under GE increased with rising MAP, with an average rate of 0.82 Mg C ha^−1^ yr^−1^ (Figure 5a). Specifically, in MAP > 500 mm regions, GE exhibited the highest soil carbon sequestration rate among all grassland restoration management treatments. In regions with 300 mm < MAP < 500 mm, the soil carbon sequestration rate of GE was second only to sowing grass and sowing legumes. Even in MAP < 300 mm regions, the soil carbon sequestration rate of GE remained similar to that of sowing legumes, fertilization, high diversity, and medium diversity, while outperforming low diversity and improved grazing (Figure 5a). When comparing the costs of common grassland management practices, GE had the lowest cost at just 33.26 USD ha^−1^, while sowing costs were approximately 87.53 USD ha^−1^. The cost of diversity control was 519.16 USD ha^−1^, and topsoil removal reached a staggering 16280.70 USD ha^−1^ (Figure 5b). In summary, among different grassland restoration management treatments, GE had a soil carbon sequestration rate second only to sowing grass, and it also had the lowest cost. Given that sowing grass typically needs to be combined with GE in practical implementation, it is reasonable to conclude that GE, as a stand‐alone measure, currently offers the best solution for achieving both effective soil carbon sequestration and minimal economic cost.

## 3 Discussion

Our meta‐analysis provides the first comprehensive, data‐driven framework for optimizing GE duration to maximize SOC sequestration in degraded grasslands. Through synthesizing an extensive dataset of paired observations spanning China's grassland ecosystems, we demonstrate that GE significantly enhances SOC content (34.1%) and stock (24.6%), with recovery dynamics strongly modulated by MAP (Figure 1). While our findings are derived from China's grasslands—a globally representative system for GE policy implementation, these findings provide critical insights for SOC sequestration strategies in arid and semi‐arid grasslands worldwide.

We found that SOC sequestration exhibited a logarithmic growth trend with increasing GE duration (Figure 2a, b), reflecting the nonlinear dynamics of ecosystem recovery. This trajectory likely emerges from coupled plant‐soil‐microbial feedbacks [30], where initial rapid carbon gains transition to stabilized accumulation through three mechanistic phases. First, in the early stages, plant communities dominated by short‐lived species rapidly recovered aboveground and belowground productivity driven by strong functional complementarity [31, 32]. This resulted in concentrated inputs of litter and root‐derived carbon, serving as the initial driver for rapid SOC accumulation [33]. Second, the substantial influx of fresh carbon during this stage may trigger priming effects [34], stimulating the mineralization of pre‐existing SOC by microorganisms and thereby causing carbon loss [35]. Third, as the duration of GE increases, although species diversity may decline [36], the high‐coverage vegetation canopy and thick litter layer significantly improve the soil microenvironment, directly inhibiting microbial mineralization activity and thereby reducing the decomposition of SOC [37]. In this study, our results provide mechanistic insights into SOC stabilization processes of long‐term GE, the functional response of the soil microbial community may undergo a critical shift [38]. Specifically, increased microbial biomass carbon (MBC) and enzyme activities (β‐glucosidase, peroxidase, sucrase, urease, and protease) under GE (Figure S6) reflect enhanced microbial metabolism and stable carbon accumulation. Correspondingly, SOC was positively correlated with MBC and enzyme activities (β‐glucosidase, peroxidase), but negatively correlated with microbial biomass nitrogen (MBN), pH, and bulk density (BD) (Figure S7). These relationships strongly suggest that enhanced microbial enzyme activities facilitate ongoing cellulose decomposition, converting plant carbon into stabilized SOC [39, 40, 41], while concurrently suppressing the decomposition of recalcitrant carbon due to a nitrogen‐limited microbial environment [42]. Under nitrogen‐limited conditions, microbial communities preferentially synthesize and accumulate stable microbial‐derived carbon, aligning well with recent global evidence highlighting microbial carbon‐use efficiency as a key determinant of SOC persistence in restored ecosystems [43]. This phased mechanism reconciles contradictory findings in restoration ecology by demonstrating how transient priming effects become subordinate to long‐term stabilization processes – a pattern recently observed in global meta‐analyses [44], but previously unresolved at ecosystem scales.

Our approach reveals that critical limitations of conventional duration‐classification methods in GE studies, demonstrating how arbitrary categorization obscures nonlinear SOC dynamics [9]. We establish 10 years as the national threshold for reaching the mean SOC recovery benchmark, while the recovery trajectory continues to decelerate and approaches an asymptotic plateau beyond ∼30 years [45, 46]. The logarithmic recovery pattern explains why discrete duration categories (e.g., “short/long‐term”) fail to capture the accelerated gains in the early stages and asymptotic convergence post‐10 years. Importantly, precipitation mediates recovery efficiency across various grassland ecosystems [47, 48]. High‐precipitation regions (> 500 mm) achieve an average SOC recovery in just 2.4 years, whereas arid zones (< 300 mm) require decades (Figure 2c, d). Below a threshold of 205 mm MAP, GE fails to enhance SOC, likely due to water limitation of microbial activity and lower plant inputs [49]. The average recovery metric provides actionable policy guidance where saturation models fail for climate‐adaptive grassland utilization.

**FIGURE 3:**
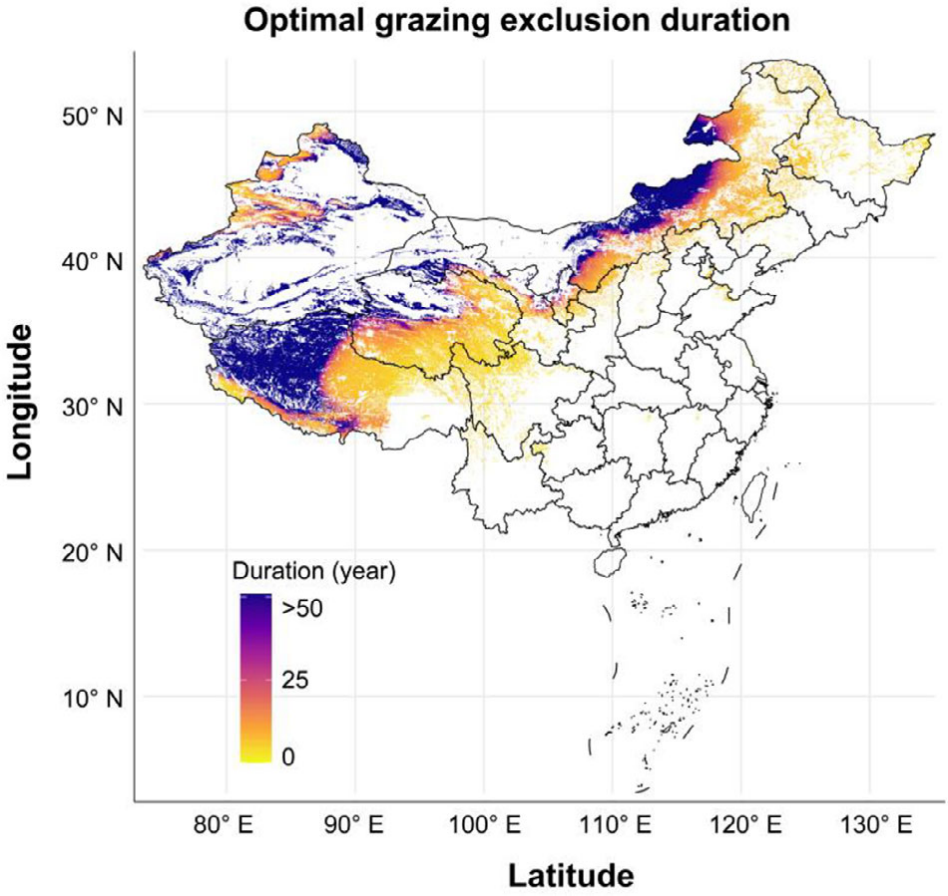
Spatial distribution of optimal grazing exclusion durations for achieving the average soil organic carbon recovery effect across grasslands in China.

Having quantified the average optimal GE duration and established that this duration varies regionally with MAP, we constructed a national‐scale spatial map of optimal GE durations (Figure 3) derived from our GE duration–MAP response model. This map directly matches SOC recovery targets with management timelines in a spatially explicit manner. Notably, our findings challenge previous studies that government‐implemented GE on the Tibetan Plateau has induced grassland degradation [50]. Our optimal GE duration map also identifies regions requiring supplementary measures (such as targeted reseeding, soil nutrient amendments where appropriate, and water‐conservation or water‐harvesting practices) beyond GE for more effective carbon sequestration, including northern Inner Mongolia, southern Tianshan Mountains (Xinjiang), and northwestern Tibetan Plateau [51]. Furthermore, in more humid southeastern zones, shortening GE durations could balance ecological and socioeconomic benefits [52]. Additionally, the optimal GE duration map can also explain the divergent results of SOC increase or decrease in previous single‐site experiments within the same region [53, 54, 55], which may be due to differences in MAP among experimental sites.

**FIGURE 4:**
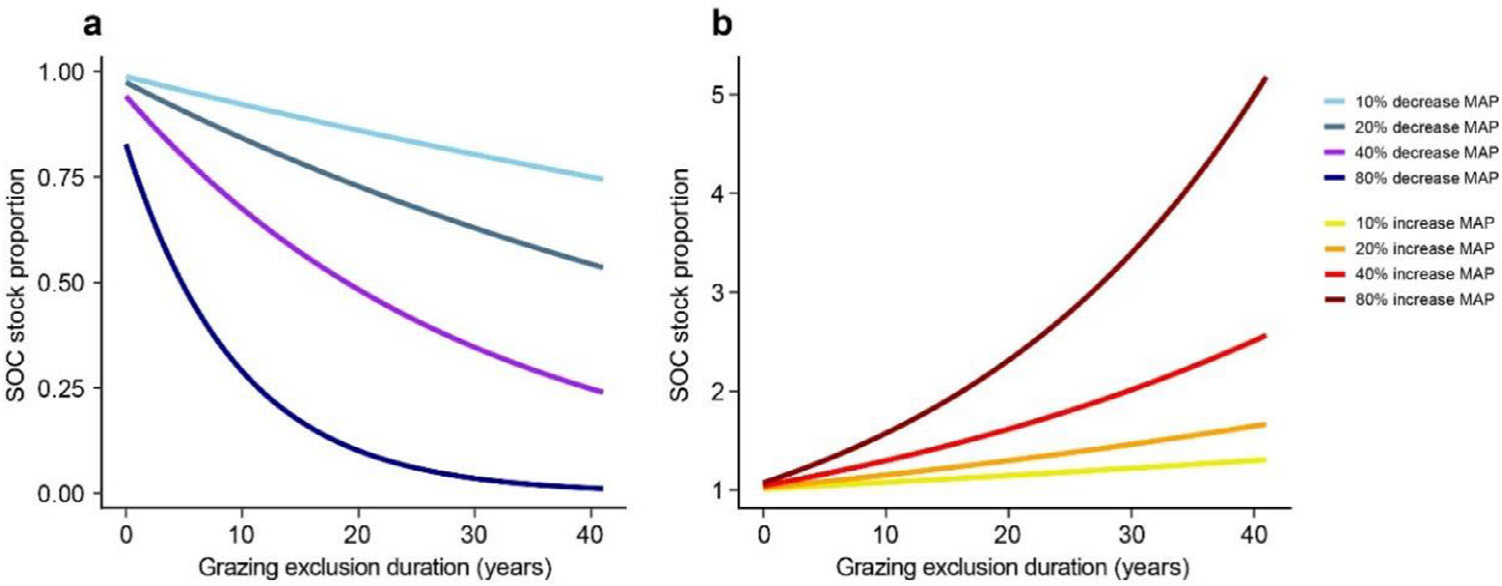
Predicted responses of soil organic carbon stock to a range of mean annual precipitation (MAP) increases and decreases. (**a)** Soil organic carbon stock response to decreases in mean annual precipitation. (**b)** Soil organic carbon stock response to increases in mean annual precipitation. The color gradient from dark to light represents different amplitudes of precipitation fluctuation, corresponding to 80%, 40%, 20%, and 10% of the mean annual precipitation. Source data for these predictions are available in the accompanying Source Data file.

Given the dominant role of MAP in modulating GE effectiveness and the future climate uncertainty [56], we simulated SOC sequestration potential under varying precipitation scenarios (Figure 4). This model reveals a nonlinear amplification or suppression of GE‐induced SOC changes across MAP change. For instance, a 40% MAP reduction decreased GE's carbon sequestration effectiveness by 18.65% at 10 years and 41.87% at 30 years, while a 40% increase boosted it by 14.56% at 10 years (reaching 26.81% under an extreme 80% increase). These findings provide a new perspective for the formulation of the current GE management strategy [24, 57, 58], and prove the necessity of dynamically adjusting the GE duration according to the expected MAP trend. Our models predict that persistently low MAP (e.g., arid northwest China, Central Asia [59, 60]) will lead to inefficient SOC recovery under fixed GE durations. Conversely, regions with increasing MAP (e.g., high‐latitude and humid grasslands) will see enhanced SOC restoration potential, although requiring vigilance against associated risks like nutrient leaching and soil degradation [61, 62].

**FIGURE 5:**
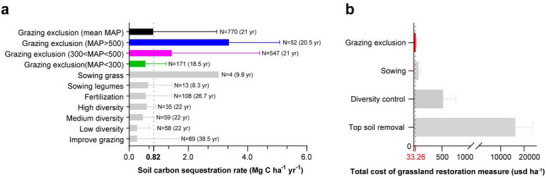
Comparison of restoration measures for degraded grasslands. (**a)** Soil organic carbon sequestration rate. (**b)** Economic cost input of various treatments. The number of studies used for calculating the average is given for each type of restoration measure. The average study duration (years) for each type of restoration measures is indicated in parentheses.

Our analysis reveals a substantial potential for SOC sequestration through nationwide GE implementation in China's degraded grasslands. Assuming an average achievable SOC sequestration rate of 0.82 Mg C ha^−^^1^ yr^−^^1^ over the GE duration (10 years; Figure 5a), extending GE to all degraded grasslands (70% of 264.5 million ha) [5] would sequester 1.52 Pg C within the topsoil layer (∼0–20 cm). This represents approximately 5% of the national grassland SOC stock (estimated at 31.2 Pg, 96% belowground) [63] and is equivalent to offsetting 17% of annual global fossil fuel emissions [64]. Given China's nationwide implementation of GE projects since the 1970s [65], our findings provide potential support that the national “Returning Grazing Land to Grassland” program contributes to global climate change mitigation through enhanced soil carbon sequestration.

Although GE aids SOC restoration in grasslands, its application would be constrained if the associated costs were excessively high [66]. By integrating and accounting for the costs of various restoration measures for degraded grasslands [44, 67, 68, 69, 70, 71, 72, 73, 74], we found that GE is the most cost‐effective option for achieving SOC restoration in grasslands, with economic costs only ranging from 2.63 to 489.50 times less than those of sowing and topsoil removal measures (Figure 5b). For decision‐makers' macro strategy planning, this result aids in the rational allocation of financial expenditures for grassland restoration management treatments, such as implementing single GE management in wet areas to reduce expenditures and investing the saved funds in arid areas to accelerate SOC restoration in degraded grasslands by adding other restoration measures based on GE. Furthermore, leveraging our validated optimal GE duration and quantitative SOC restoration dynamics model, the predicted grassland SOC recovery enables pioneering integration of GE‐derived carbon credits into China's national CCER (Chinese Certified Emission Reduction) system [75], with future pathways to international carbon markets. Establishing grassland carbon certification and trading mechanisms led by herder cooperatives, where a defined majority of revenue directly compensates pastoralists in GE zones, will accelerate global restoration of degraded grasslands.

Our findings redefine GE as a dynamic, climate‐adaptive tool by quantifying the nonlinear GE duration–MAP interaction. These insights are immediately actionable for policymakers, particularly in dryland regions where grassland degradation threatens both ecosystem stability and food security. Although MAP×MAT and MAP×pH were not supported by AICc‐based model selection, regressions of SOC stock response on environmental and plant traits imply that hydrothermal and edaphic contexts can still interact with water availability to modulate plant inputs and microbial turnover, contributing to longer‐term heterogeneity in SOC recovery under GE (Table S7 and Figure S7). Future research should clarify how GE‐induced shifts in soil microbial communities and extracellular enzyme activities regulate soil carbon stabilization. Integrating these mechanistic insights with socio‐economic considerations will enhance the effectiveness, equity, and sustainability of grazing exclusion strategies. Additionally, it is crucial to identify synergistic optimization pathways between SOC restoration and forage production. These pathways can enhance the dynamic updating and spatial adaptability of predictive models, thereby improving their accuracy and applicability across different regions. By bridging mechanistic understanding and scalable policy design, our work transforms GE from a blanket restoration tool into a precision instrument for climate‐smart grassland management‐a model applicable to global drylands facing similar degradation pressures.

## 4 Methods

### 4.1 Data Collection

We systematically searched all peer‐reviewed publications that were published before June 2024 that investigated the effects of grazing exclusion on SOC using the Web of Science (Core Collection; http://www.webofknowledge.com), Google Scholar (http://scholar.google.com) and the China National Knowledge Infrastructure (CNKI; https://www.cnki.net) with the search term (“soil organic carbon” OR SOC OR “soil carbon storage” OR “soil carbon pool” OR “soil carbon stock” OR “soil carbon concentration”) AND (“grazing exclusion” OR fencing OR enclosure OR fenced OR enclosed OR “forbidding grazing” OR “grazing prohibition” OR enclosing OR fence). We also searched for references within these papers. The literature search was performed following the guidelines of PRISMA (Figure S1) (Preferred Reporting Items for Systematic Reviews and Meta‐Analyses) [76].

We employed the following criteria to select the studies: (1) they were purposely designed to test the effects of GE on SOC (both SOC content and stock); (2) they had at least three replicated GE treatments and a grazing treatment as control; they had the same initial climatic and soil properties in the GE and grazing treatment plots. To better represent responses of SOC under natural conditions, we did not include greenhouse and mesocosm studies. In total, 125 publications met these criteria (Table S1). Multiple independent experiments were conducted at different locations, which were considered separate studies. When the same data was included in different publications, we only recorded the data once. If a study included different grazing restrictions, we considered it as independent observation results. For each site, we extracted the means, the number of replications, and the standard deviations of SOC content, SOC stock, AGB, BGB, TN, TP, pH, BD, MBC, MBN, β‐glucosidase, peroxidase, sucrase, urease, and protease, if reported.

When an original study reported the results graphically, we used WebPlotDigitizer (https://apps.automeris.io) to extract data from the figures. We also extracted GE duration, soil depth, grassland type (alpine, typical, desert and meadow), latitude, longitude, MAT (°C) and MAP (mm), if reported) from original or cited papers, or cited data sources, when MAT unavailable, it was derived based on geographic locations using the WorldClim version 2 dataset [77]. The GE duration refers to the time interval in grassland, from the establishment of fencing and the start of GE to the measurement data recorded in the literature. The soil depth was recorded as the midpoint of each soil‐depth interval [78]. Grassland type (L) was extracted as reported in each primary study and harmonized into four broad zonal ecosystem categories (alpine, meadow, typical, desert) [79]; it was evaluated as a potential moderator via AIC‐based model selection and complementary mixed‐effects tests (Table S2 and Figure S2).

In this study, “degraded grasslands” refers to rangeland systems that have experienced a persistent decline in ecological condition and/or productivity, typically manifested as reduced vegetation cover or biomass, increased bare ground and erosion risk, and/or losses of soil fertility and SOC under long‐term anthropogenic pressures (most commonly overgrazing). We operationalized this definition using the descriptions provided in the primary studies: sites were considered degraded when the original articles explicitly characterized them as degraded/overgrazed/desertified (or equivalent terms) and/or when GE (fencing) was implemented as a restoration measure to reverse degradation symptoms. Because quantitative and comparable degradation‐severity metrics (e.g., grazing history, vegetation‐cover deficit relative to a reference state, or SOC deficit) were not consistently reported across the compiled studies, we did not standardize degradation intensity across sites; instead, degradation served as an eligibility criterion, and we interpret our estimates as average responses across degraded grasslands as defined by the primary literature.

### 4.2 Data Analysis

The natural log‐transformed response ratio (ln*RR*) was employed to quantify the effects of GE following [80]:

(1) $$\ln RR=\ln\left(\frac{{\bar{X}}_{t}}{{\bar{X}}_{c}}\right)=\ln{\bar{X}}_{t}\;-\ln{\bar{X}}_{c}$$

where ${\bar{X}}_{t}$ and ${\bar{X}}_{c}$ are the observed values of a selected variable in the GE and the expected value of the grazing in each study, respectively.

In our dataset, standard deviation or standard error was reported in all 125 publications. Like in previous studies, we employed the number of replications for weighting [81]:

(2) $$W_{r}=\left(N_{c}\times N_{t}\right)/\left(N_{c}+N_{t}\right)$$

where *Wr* is the weight associated with each ln*RR* observation, and *N_c_* and *N_t_* are the number of replications in grazing and GE, respectively.

For model fitting, MAP and GE duration were not used to compute ln*RR*; instead, they were extracted as continuous moderators associated with each ln*RR* observation and evaluated in mixed‐effects meta‐regression, to visualize precipitation‐dependent trajectories, we additionally grouped MAP into three commonly used classes (< 300, 300–500, > 500 mm) and plotted model‐predicted lnRR curves using representative values within each class. SOC content, SOC stock, AGB, BGB, TN, TP, pH, and BD were considered as our response variables and analyzed separately. Across the compiled studies, soil pH covered a broad gradient (grazed controls: 5.50–10.38, mean ± SD = 8.10 ± 0.86; GE plots: 5.50–10.24, mean ± SD = 7.92 ± 0.85). To validate the linearity assumption for the continuous predictors, we first graphically plotted the ln*RR* versus individual predictors and identified logarithmic functions as an alternative to linear functions. We also statistically compared the linear and logarithmic functions with the predictor of interest as the fixed effect, and ‘study’ as the random effect, using the Akaike information criterion (AIC).

For each environmental attribute, we tested whether its response to GE differed from zero and whether ln*RR* was affected by MAP (P, mm), GE duration (T, years), and environmental variables (*E*, that is, grassland type, MAT, or soil depth) using the following model:

(3) $$\begin{array}{ccc} & & \ln RR=\beta_{0}+\beta_{1}\cdot P+\beta_{2}\cdot T+\beta_{3}\cdot P\times T+\beta_{4}\cdot E+\beta_{5}\cdot P\times E+\\ & & \beta_{6}\cdot T\times E+\beta_{7}\cdot P\times T\times E+\pi_{\mathrm{study}}+\varepsilon \end{array}$$

where *β* is the coefficient to be estimated; π_study_ is the random effect factor of study, accounting for the autocorrelation among observations within each study; *ɛ* is sampling error. We conducted the analysis using restricted maximum likelihood estimation with the *lme4* package with *Wr* as the weight for each corresponding observation [82]. To prevent overfitting [83], we selected the most parsimonious model among all alternatives with the condition to keep *P* and *T*, as they were part of our core hypotheses to be tested. The model selection was accomplished by using the ‘dredge’ function of the *MuMIn* package [84]. All terms associated with grassland type (Equation 3) were excluded in the most parsimonious models (Table S2). The most parsimonious models were selected based on the lowest AIC values, considering models with ΔAIC < 2 as equivalently supported. To further examine the effects of grassland types (*L*), we conducted an analysis with *L* as the only fixed factor and study as the random factor, and the analysis confirmed that there was no difference in the environmental attributes among grassland types except AGB and BGB (Table S2). We assessed the assumption of linearity between ln*RR* and continuous predictors by comparing near and log‐linear responses. Natural log transformed *P*, ln(*P*), yielded lower or similar AIC values than *P*, whereas *T* was better than ln(*T*) (Table S3).

We scaled all continuous predictors (observed values minus the mean and divided by one standard deviation). When continuous predictors are scaled, *β_0_* is the overall mean ln*RR* at the mean ln(*P*) and mean ln(*T*) [84]. To graphically illustrate whether the effect of the GE on ln*RR* differed with MAP, we calculated MAP‐dependent effects using the recommended scope and the range of MAP in our dataset by dividing the MAP into the following categories: MAP < 300 mm, 300 mm < MAP < 500 mm, and MAP > 500 mm.

We examined whether MAT and soil depth of study sites affected the responses of environmental attributes to the effects of the GE duration and MAP by substituting *E* in Equation (3) by MAT and soil depth, respectively. Similarly, we selected the most parsimonious models using the method described above. All terms associated with MAT and soil depth were excluded during the model selection, except for soil bulk density to soil depth. The *E* in Equation (3) was individually modeled for two reasons. First, these variables are inherently correlated, and simultaneous modeling would lead to strong multicollinearity. Second, simultaneous modeling would include a large number of predictors, greater than the number of studies in our metadata.

For the final models of SOC content and stock, total nitrogen, total phosphorus, soil pH, and bulk density, all terms associated with *E* (Equation (3)) were excluded, but the final models of AGB and BGB were included with grassland type (*L*). The model selection led to Equation (4) for AGB and BGB and Equation (5) for SOC content and stock, total nitrogen, total phosphorus, soil pH, and bulk density as the most parsimonious models, respectively:

(4) $$\begin{array}{ccc} & & \ln RR=\beta_{0}+\beta_{1}\cdot P+\beta_{2}\cdot T+\beta_{3}\cdot P\times T+\beta_{4}\cdot P\\ & & \times L+\beta_{5}\cdot T\times L+\pi_{\mathrm{study}}+\varepsilon \end{array}$$

(5) $$\ln RR=\beta_{0}+\beta_{1}\cdot In\left(P\right)+\beta_{2}\cdot T+\beta_{3}\cdot P\times T+\pi_{\mathrm{study}}+\varepsilon$$

We employed a mixed‐effects model funnel plot asymmetry Egger regression test to assess the likelihood of publication bias influencing the results, using sample size as a predictor. The Egger test was conducted on our primary statistical analyses (the response ratio for the entire dataset, as well as the response ratio incorporating the relevant covariates from Equations (4) and (5)). With the exception of BGB and total phosphorus, no significant publication bias was detected, which could potentially deviate our results from the significant effects of the Egger regression (Table S4). Multicollinearity among explanatory variables was examined by evaluating the variance inflation factor (only models with all predictors' variance inflation factors less than 5 were accepted) [85], and no issues of multicollinearity were found in the most simplified models (Table S5).

For ease of interpretation, ln*RR* and its corresponding 95% confidence intervals (CIs) were transformed into a percentage change between GE and grazing as [86]:

(6) $$\left(\begin{array}{c} e^{\ln RR}-1 \end{array}\right)\times 100\%$$

If the CIs did not cover zero, the effect between GE and grazing on environmental attributes differed significantly at α = 0.05.

To illustrate the effects of GE on environmental attributes over time, we compared the ln*RR* when the MAP was *R_1_* (no increase or decrease of MAP) and *R_α_* (α % increase or decrease of MAP). We assumed that the mean value of GE duration, *X_c_*, did not vary with MAP, which led to the following equation:

(7) $$P_{\alpha }={\left(R_{\alpha }/R_{1}\right)}^{\beta_{1}+\beta_{3}\cdot T}$$

where *P_α_* is the proportion of remaining SOC under *α*% higher or lower MAP in a period of *T*, and other model terms were described in Equation (3). The detailed derivation process for Equation (5) is presented in Supplementary Methods. Based on Equation (5), we fitted curves for the increase or decrease in SOC over time when there was a 10, 20, 40, and 80% increase or decrease in MAP.

We evaluated the potential influence of spatial autocorrelation on our model by performing Moran's *I* tests on the residuals from Equation (4), using the R package ‘*ape*’ [87]. The results indicated no significant spatial autocorrelation in any of the regression models (Table S6 and Figure S8).

To map optimal GE duration at the national scale (Figure 3), we used a benchmark‐based operational definition. We first defined the benchmark as the nationwide average SOC stock recovery effect (24.79%). Specifically, we first obtained annual precipitation raster data for China from 2000 to 2023 from the National Tibetan Plateau Data Center (https://www.tpdc.ac.cn/zh‐hans/data/faae7605‐a0f2‐4d18‐b28f‐5cee413766a2), and then calculated the spatial distribution of the multi‐year MAP via raster calculations. Using the AIC‐best SOC stock model that includes MAP, GE duration, and their interaction, we then inversely solved the fitted response surface to obtain, for each MAP value, the minimum GE duration required to reach this benchmark. These spatially explicit required durations were calculated from gridded MAP data and mapped across China.

About 50.65% (n  =  390 of 770) measurements provided SOC stock data. For the remaining data, SOC stocks were converted from SOC content (n  =  380). SOC content data were converted to stocks based on soil bulk density and soil depth. Especially, when data on bulk density were not provided (n  = 388), we estimated them based on empirical relationships between SOC content (SOC; g kg^−1^) and bulk density (BD; g cm^−3^) across the reported data for GE and grazed sites (Equations (8) and (9) and Figure S5) [88]:

(8) $$BD_{\mathrm{grazing}\;}=0.9546\times e^{-0.0121\mathrm{SOC}}+0.5651,\;P<0.001$$

(9) $$BD_{\mathrm{grazing}\;\mathrm{exclusion}}=0.7322\times e^{-0.0186\mathrm{SOC}}+0.7288,P<0.001$$

In order to compare the carbon sequestration rates of different grassland management strategies, we acquired the relevant data on sowing grass, sowing legumes, fertilization, diversity control, and improved grazing (i.e. Rotational grazing) from previous literature via Webplotdigitizer (V4.8) and compared them with the carbon sequestration rates of GE in different MAP regions in this study. Meanwhile, by obtaining the economic input data of grassland restoration measures in previous literature, we compared the economic costs of different grassland restoration measures. We also tested whether the responses to GE differed with soil depth, and we found consistent responses across all sampling depths (Figure S4). All analyses were performed in R 4.4.2 [89].

## Conflicts of Interest

The authors declare no conflicts of interest.

## Supporting information




**Supporting File**: advs74783‐sup‐0001‐SuppMat.docx.

## Data Availability

The data that support the findings of this study are available from the corresponding author upon reasonable request.

## References

[1] J. Ling , J. A. J. Dungait , M. Delgado‐Baquerizo , Z. Cui , et al., “Soil Organic Carbon Thresholds Control Fertilizer Effects on Carbon Accrual in Croplands Worldwide,” Nature Communications 16 (2025): 3009. 10.1038/s41467-025-57981-6 PMC11950326 40148281

[2] Z. Zhou , C. Wang , Y. Li , X. Wang , X. He , M. Xu , and A. Cai , “Carbon Gain in Upper but Loss in Deeper Cropland Soils Across China Over the Last Four Decades,” Proceedings of the National Academy of Sciences 122 (2025): 2422371122. 10.1073/pnas.2422371122 PMC11725835 39739784

[3] Y. Bai and M. F. Cotrufo , “Grassland Soil Carbon Sequestration: Current Understanding, Challenges, and Solutions,” Science 377 (2022): 603–608. 35926033 10.1126/science.abo2380

[4] S. Luo , G. K. Png , N. J. Ostle , H. Zhou , et al., “Grassland Degradation‐induced Declines in Soil Fungal Complexity Reduce Fungal Community Stability and Ecosystem Multifunctionality,” Soil Biology and Biochemistry 176 (2023): 108865.

[5] P. Qingmin , Y. Yuanhe , and H. Jianhui , “Limiting Factors of Degraded Grassland Restoration in China and Related Basic Scientific Issues,” Bulletin of National Natural Science Foundation of China 37 (2023): 571–579.

[6] Y. Hu , T. Kou , M. Cong , Y. Jia , et al., “Grassland Degradation‐induced Soil Organic Carbon Loss Associated with Micro‐food Web Simplification,” Soil Biology and Biochemistry 201 (2025): 109659.

[7] J. Sun , Y. Wang , T. M. Lee , X. Nie , et al., “Nature‐based Solutions can Help Restore Degraded Grasslands and Increase Carbon Sequestration in the Tibetan Plateau,” Communications Earth & Environment 5 (2024): 154.

[8] Q. Qu , L. Deng , A. Gunina , X. Hai , J. Deng , Z. Shangguan , and Y. Kuzyakov , “Grazing Exclusion Increases Soil Organic C through Microbial Necromass of Root‐derived C as Traced by ^13^C Labelling Photosynthate,” Biology and Fertility of Soils 60 (2024): 407–420.

[9] Z. Hu , S. Li , Q. Guo , S. Niu , N. He , L. Li , and G. Yu , “A Synthesis of the Effect of Grazing Exclusion on Carbon Dynamics in Grasslands in China,” Global Change Biology 22 (2016): 1385–1393. 26485056 10.1111/gcb.13133

[10] L. Deng , Z.‐P. Shangguan , G.‐L. Wu , and X.‐F. Chang , “Effects of Grazing Exclusion on Carbon Sequestration in China's Grassland,” Earth‐Science Reviews 173 (2017): 84–95.

[11] D. Beillouin , M. Corbeels , J. Demenois , D. Berre , et al., “A Global Meta‐Analysis of Soil Organic Carbon in the Anthropocene,” Nature Communications 14 (2023): 3700. 10.1038/s41467-023-39338-z PMC10287672 37349294

[12] G. Zhang , X. Tan , J. He , D. Luo , et al., “Grazing Exclusion Promotes Soil Organic Carbon Accumulation in Tibetan Grasslands with Lower Temperatures,” Ecological Processes 13 (2024): 79.

[13] Y. Shen , Y. Fang , H. Chen , Z. Ma , et al., “New Insights into the Relationships between Livestock Grazing Behaviors And Soil Organic Carbon Stock in an Alpine Grassland,” Agriculture, Ecosystems & Environment 355 (2023): 108602.

[14] C. Du , J. Jing , Y. Shen , H. Liu , and Y. Gao , “Short‐term Grazing Exclusion Improved Topsoil Conditions and Plant Characteristics in Degraded Alpine Grasslands,” Ecological Indicators 108 (2020): 105680.

[15] Y. Qi , D. Wei , Z. Wang , H. Zhao , J. Fan , J. Tao , and X. Wang , “Optimizing Restoration Duration to Maximize CO_2_ Uptake on the Tibetan Plateau,” Catena 241 (2024): 108060.

[16] J. Cui , Y. Li , J. F. Adamowski , J. Cao , A. Biswas , J. Wang , and X. Zhang , “Response of leaf, litter, and root ecological stoichiometries to grazing exclosure duration on the Qinghai‐Tibetan Plateau,” Soil and Tillage Research 241 (2024): 106123.

[17] C. E. Kazanski , J. Cowles , S. Dymond , A. T. Clark , et al., “Water Availability Modifies Productivity Response to Biodiversity and Nitrogen in Long‐term Grassland Experiments,” Ecological Applications 31 (2021): 02363. 10.1002/eap.2363 33899307

[18] Z. Li , F. Wang , F. Su , P. Wang , et al., “Climate change Drivers Alter Root Controls Over Litter Decomposition In A Semi‐Arid Grassland,” Soil Biology and Biochemistry 158 (2021): 108278.

[19] H. He , J. Zhou , Y. Wang , et al., “Deciphering Microbiomes Dozens of Meters Under Our Feet and their Edaphoclimatic and Spatial Drivers,” Global Change Biology 30 (2024): 17028. 10.1111/gcb.17028 37955302

[20] J. Su , Y. Zhao , F. Xu , and Y. Bai , “Multiple Global Changes drive Grassland Productivity and Stability: A Meta‐analysis,” Journal of Ecology 110 (2022): 2850–2869.

[21] L. Xiao , M. Leng , P. Greenwood , R. Zhao , Z. Xie , Z. You , and J. Liu , “Temporal and Vertical Dynamics of Carbon Accumulation Potential Under Grazing‐Excluded Grasslands in China: The Role of Soil Bulk Density,” Journal of Environmental Management 351 (2024): 119696. 38042080 10.1016/j.jenvman.2023.119696

[22] J. Cao , L. Wang , J. F. Adamowski , A. Biswas , M. R. Alizadeh , and Q. Feng , “A Context‐dependent Response of Soil Carbon and Nitrogen to Grazing Exclusion: Evidence from a Global Meta‐analysis,” Journal of Cleaner Production 434 (2024): 139792.

[23] H. Wang , X. Li , C. Pang , H. Y. H. Chen , et al., “Contrasting Effects of Short‐ and Long‐term Grazing Exclusion on Plant Diversity in Humid Grasslands,” Agriculture, Ecosystems & Environment 381 (2025): 109420.

[24] H. Wang , Y. Li , Y. He , H. Y. H. Chen , et al., “Grazing Exclusion Facilitates more Rapid Ecosystem Carbon Sequestration of Degraded Grasslands in Humid than in Arid regions,” Agriculture, Ecosystems & Environment 353 (2023): 108553.

[25] J. Li , Z. Shangguan , and L. Deng , “Free Particulate Organic Carbon Plays Critical Roles in Carbon Accumulations During Grassland Succession Since Grazing Exclusion,” Soil and Tillage Research 220 (2022): 105380.

[26] Z.‐Q. Yuan , X. Song , Z. Feng , J. Wang , et al., “Soil Organic Carbon and Nitrogen Sequestration Following Grazing Exclusion on the Loess Plateau, China,” Catena 232 (2023): 107412.

[27] C. Peng , L. Shi , Y. He , Z. Yao , et al., “Climate Factors Regulate the Depth Dependency of Soil Organic Carbon Under Grazing Exclusion In Chinese Grasslands: A Meta‐Analysis,” Land Degradation & Development 34 (2023): 4924–4934.

[28] S. Zhou , Y. Dong , H. Yang , S. Yang , et al., “Effects of Grazing Exclusion on Soil Properties, Fungal Community Structure, and Diversity in Different Grassland Types,” Ecology and Evolution 14 (2024): 11056. 10.1002/ece3.11056 PMC10905231 38435014

[29] X. Tai , H. E. Epstein , and B. Li , “Effects of Grazing Exclusion on Spring and Autumn Pastures in Arid Regions of China: Insights from Field Surveys and landsat Images,” Agriculture, Ecosystems & Environment 310 (2021): 107302.

[30] Y. Yang , Y. Dou , B. Wang , Y. Wang , et al., “Increasing Contribution of Microbial Residues to Soil Organic Carbon in Grassland Restoration Chronosequence,” Soil Biology and Biochemistry 170 (2022): 108688.

[31] Y. Gao , Y. An , B. Qi , J. Liu , H. Yu , and D. Wang , “Grazing Exclusion Mediates the Trade‐Off Between Plant Diversity and Productivity in Leymus Chinensis Meadows along a Chronosequence on the Songnen Plain, China,” Ecological Indicators 126 (2021): 107655.

[32] S. Yin , X. Chen , G. Piton , C. Terrer , et al., “The Complementarity Hypothesis Reversed: Root Trait Similarity in Species Mixtures Promotes Soil Organic Carbon in Agroecosystems,” Soil Biology and Biochemistry 203 (2025): 109736.

[33] Y. Li , K. Buckeridge , B. Wang , Q. Huang , et al., “Grazing Exclusion Enhanced the Capability of Soil Microorganisms to Access Photosynthetic Carbon in Loess Plateau Grassland,” Soil Biology and Biochemistry 203 (2025): 109743.

[34] J. Feng and B. Zhu , “Global Patterns and Associated Drivers of Priming Effect in Response to Nutrient Addition,” Soil Biology and Biochemistry 153 (2021): 108118.

[35] C. Li , C. Xiao , M. Li , L. Xu , and N. He , “The Quality and Quantity of SOM Determines the Mineralization of Recently added labile C and Priming of Native SOM in Grazed Grasslands,” Geoderma 432 (2023): 116385.

[36] L. Yu , W. Sun , H. Zhang , N. Cong , Y. Chen , J. Hu , and X. Jing , “Grazing Exclusion Jeopardizes Plant Biodiversity Effect but Enhances Dryness Effect on Multifunctionality in Arid Grasslands,” Agriculture, Ecosystems & Environment 363 (2024): 108883.

[37] Y. Liu , C. Wang , L. Xu , and N. He , “Effect of Grazing Exclusion on the Temperature Sensitivity of Soil Net Nitrogen Mineralization in the Inner Mongolian Grasslands,” European Journal of Soil Biology 97 (2020): 103171.

[38] J. Shi , L. Lu , J. Zang , Y. Sun , et al., “Multitrophic interactions support belowground carbon sequestration through microbial necromass accumulation in dryland biocrusts,” Soil Biology and Biochemistry 202 (2025): 109708.

[39] Z. Wu , Z. Tang , T. Yu , J. Zhang , et al., “Nitrogen Fertilization Rates Mediate Rhizosphere Soil Carbon Emissions of Continuous Peanut Monoculture by Altering Cellulose‐specific Microbess,” Frontiers in Plant Science 14 (2023): 1109860. 36938001 10.3389/fpls.2023.1109860 PMC10021708

[40] J. Gong , X. Dong , X. Li , K. Yue , et al., “Phosphorus Fertilization Affects Litter Quality And Enzyme Activity In A Semiarid Grassland,” Plant And Soil 492 (2023): 91–108.

[41] G. Liang , J. Stark , and B. G. Waring , “Mineral Reactivity Determines Root Effects On Soil Organic Carbon,” Nature Communications 14 (2023): 4962. 10.1038/s41467-023-40768-y PMC10432558 37587139

[42] M. J. Woods , G. K. Attea , and R. W. McEwan , “Resprouting of the Woody Plant Pyrus Calleryana Influences Soil Ecology During Invasion Of Grasslands in the American Midwest,” Applied Soil Ecology 166 (2021): 103989.

[43] F. Tao , Y. Huang , B. A. Hungate , S. Manzoni , et al., “Microbial Carbon Use Efficiency Promotes Global Soil Carbon Storage,” Nature 618 (2023): 981–985. 37225998 10.1038/s41586-023-06042-3 PMC10307633

[44] P. Török , E. Vida , B. Deák , S. Lengyel , and B. Tóthmérész , “Grassland Restoration on Former Croplands in Europe: An Assessment of Applicability of Techniques and Costs,” Biodiversity and Conservation 20 (2011): 2311–2332.

[45] L. Wang , Y. Wang , D. Sun , J. Wang , S.‐J. Lee , R. A. Viscarra Rossel , and Y. Gan , “Soil Carbon Stocks in Temperate Grasslands Reach Equilibrium With Grazing Duration,” Science of The Total Environment 949 (2024): 175081. 39069182 10.1016/j.scitotenv.2024.175081

[46] C. Wang , N. He , J. Zhang , Y. Lv , and L. Wang , “Long‐Term Grazing Exclusion Improves the Composition and Stability of Soil Organic Matter in Inner Mongolian Grasslands,” PLoS ONE 10 (2015): 0128837. 10.1371/journal.pone.0128837 PMC4461302 26057249

[47] M. A. Denboba , “Grazing management and carbon sequestration in the Dry Lowland Rangelands of Southern Ethiopia,” Sustainable Environment 8 (2022): 2046959.

[48] D. Arije , R. Ghimire , P. Bista , S. V. Angadi , and C. C. Gard , “Soil Organic Carbon Recovery and Soil Health in Semi‐arid Drylands with Years of Transition to Perennial Grasses,” Journal of Arid Environments 225 (2024): 105263.

[49] M. D. Smith , K. D. Wilkins , M. C. Holdrege , et al., “Extreme Drought Impacts have been Underestimated in Grasslands and Shrublands Globally,” Proceedings of the National Academy of Sciences 121 (2024): 2309881120. 10.1073/pnas.2309881120 PMC10823251 38190514

[50] Y. Niu , J. M. H. Knops , L. Hua , and A. Jentsch , “Flawed restoration plans on Tibetan plateau,” Science 386 (2024): 1233–1234. 10.1126/science.ads3634 39666813

[51] M. Huang , L. Ma , X. Chen , T. Zhang , R. Guo , A. A. Degen , and Z. Shang , “Grazing Stabilized Carbon and Nitrogen Pools by Reducing Carbon and Net Nitrogen Mineralization After Soil Nutrients Were Added,” Applied Soil Ecology 201 (2024): 105509.

[52] Z. Zhang , Y. Liu , J. Sun , and G.‐L. Wu , “Suitable Duration of Grazing Exclusion for Restoration of a Degraded Alpine Meadow on the Eastern Qinghai‐Tibetan Plateau,” Catena 207 (2021): 105582.

[53] M. Liu , Z. Zhang , J. Sun , Y. Wang , et al., “One‐year Grazing Exclusion Remarkably Restores Degraded Alpine Meadow at Zoige, Eastern Tibetan Plateau,” Global Ecology and Conservation 22 (2020): 00951.

[54] X. Lu , Y. Yan , J. Sun , et al., “Short‐term Grazing Exclusion has No Impact on Soil Properties and Nutrients of Degraded Alpine Grassland in Tibet, China,” Solid Earth 6 (2015): 1195–1205.

[55] X.‐M. Shi , X. G. Li , C. T. Li , Y. Zhao , Z. H. Shang , and Q. Ma , “Grazing Exclusion Decreases Soil Organic C Storage at an Alpine Grassland of the Qinghai–Tibetan Plateau,” Ecological Engineering 57 (2013): 183–187.

[56] D. Liu , C. Zhang , R. Ogaya , M. Fernández‐Martínez , T. A. M. Pugh , and J. Peñuelas , “Increasing Climatic Sensitivity of Global Grassland Vegetation Biomass and Species Diversity Correlates with Water Availability,” New Phytologist 230 (2021): 1761–1771. 33577084 10.1111/nph.17269 PMC8252445

[57] Y. Tang , C. Zhou , K. Chen , S. Xing , et al., “Grazing Exclusion Enriches Arbuscular Mycorrhizal Fungal Communities and Improves Soil Organic Carbon Sequestration in the Alpine Steppe of Northern Xizang,” Journal of Integrative Agriculture 24 (2025): 913–924, 10.1016/j.jia.2024.08.024.

[58] X. Yang , B. Wang , and S. An , “Root derived C rather than Root Biomass Contributes to the Soil Organic Carbon Sequestration in Grassland Soils with Different Fencing Years,” Plant and Soil 469 (2021): 161–172.

[59] X. Zhang , Y. Chen , G. Fang , Z. Xia , et al., “Future Changes in Extreme Precipitation from 1.0 °C more Warming in the Tienshan Mountains, Central Asia,” Journal of Hydrology 612 (2022): 128269.

[60] Q. Liang , Y. Chen , W. Duan , C. Wang , et al., “Temporal and Spatial Changes of Extreme Precipitation And Its Related Large‐Scale Climate Mechanisms in the Arid Region of Northwest China during 1961–2022,” Journal of Hydrology 658 (2025): 133182.

[61] C. Calvo , L. Rodríguez‐Gallego , G. de León , L. Cabrera‐Lamanna , et al., “Potential of Different Buffer Zones as Nature‐based Solutions to Mitigate Agricultural Runoff Nutrients in the Subtropics,” Ecological Engineering 207 (2024): 107354.

[62] E. Zhu , T. Liu , L. Zhou , S. Wang , et al., “Leaching of Organic Carbon from Grassland Soils Under Anaerobiosis,” Soil Biology and Biochemistry 141 (2020): 107684.

[63] G. Shuqin and Z. Xia , “Carbon Sequestration of Grassland in China,” Chinese Journal of Engineering Science 18 (2016): 73–79.

[64] X. Chen , H. Y. H. Chen , C. Chen , Z. Ma , E. B. Searle , Z. Yu , and Z. Huang , “Effects of Plant Diversity on Soil Carbon in Diverse Ecosystems: A Global Meta‐Analysis,” Biological Reviews 95 (2020): 167–183. 31625247 10.1111/brv.12554

[65] L. Wang , Y. Gan , M. Wiesmeier , G. Zhao , et al., “Grazing Exclusion—An Effective Approach for Naturally Restoring Degraded Grasslands in Northern China,” Land Degradation & Development 29 (2018): 4439–4456.

[66] M. L. Knight and G. E. Overbeck , “How much does is Cost to Restore a Grassland?,” Restoration Ecology 29 (2021): 13463.

[67] M. C. Resch , M. Schütz , N. Buchmann , B. Frey , et al., “Evaluating Long‐term Success in Grassland Restoration: An Ecosystem Multifunctionality Approach,” Ecological Applications 31 (2021): 02271. 10.1002/eap.2271 33615604

[68] A. Klimkowska , P. Dzierża , K. Brzezińska , W. Kotowski , and P. Mędrzycki , “Can we Balance the High Costs of Nature Restoration with the Method of Topsoil Removal? Case Study from Poland,” Journal for Nature Conservation 18 (2010): 202–205.

[69] S. Dong , Y. Xu , S. Li , H. Shen , M. Yang , and J. Xiao , “Restoration Actions Associated with Payment for Ecosystem Services Promote the Economic Returns of Alpine Grasslands in China,” Journal of Cleaner Production 458 (2024): 142439.

[70] Y. Pan , J. Wu , Y. Zhao , et al., “Comparison of the Benefits of Conservation and Forage Planting Grasslands in the River Valley Area of Tibet,” Acta Ecologica Sinica 39 (2019): 4488–4498.

[71] M. Li , S. Liu , F. Wang , H. Liu , Y. Liu , and Q. Wang , “Cost‐benefit Analysis of Ecological Restoration based on Land use Scenario Simulation and Ecosystem Service on the Qinghai‐Tibet Plateau,” Global Ecology and Conservation 34 (2022): 02006.

[72] Y. Xu , S. Dong , X. Gao , M. Yang , et al., “Trade‐offs and Cost‐benefit of Ecosystem Services of Revegetated Degraded Alpine Meadows over time on the Qinghai‐Tibetan Plateau,” Agriculture, Ecosystems & Environment 279 (2019): 130–138.

[73] R. Eschen , K. Bekele , P. R. Mbaabu , C. J. Kilawe , and S. Eckert , “ *Prosopis juliflora* Management and Grassland Restoration in Baringo County, Kenya: Opportunities for Soil Carbon Sequestration and Local Livelihoods,” Journal of Applied Ecology 58 (2021): 1302–1313.

[74] S. Schaub , R. Finger , N. Buchmann , V. Steiner , and V. H. Klaus , “The Costs of Diversity: Higher Prices for More Diverse Grassland Seed Mixtures,” Environmental Research Letters 16 (2021): 094011.

[75] L. Li , F. Ye , Y. Li , and C.‐T. Chang , “How will the Chinese Certified Emission Reduction Scheme Save Cost for the National Carbon Trading System?,” Journal of Environmental Management 244 (2019): 99–109. 31108316 10.1016/j.jenvman.2019.04.100

[76] D. Moher , A. Liberati , J. Tetzlaff , and D. G. Altman , “Preferred Reporting Items for Systematic Reviews and Meta‐analyses: The PRISMA Statement,” International Journal of Surgery 8 (2010): 336–341.20171303

[77] S. E. Fick and R. Hijmans , “WorldClim 2: New 1‐km Spatial Resolution Climate Surfaces For Global Land Areas,” International Journal of Climatology 37 (2017): 4302–4315.

[78] H. Y. H. Chen and B. W. Brassard , “Intrinsic and Extrinsic Controls of Fine Root Life Span,” Critical Reviews in Plant Sciences 32 (2013): 151–161.

[79] L. Kang , X. Han , Z. Zhang , and O. J. Sun , “Grassland Ecosystems in China: Review of Current Knowledge and Research Advancement,” Philosophical Transactions of the Royal Society B: Biological Sciences 362 (2007): 997–1008. 10.1098/rstb.2007.2029 PMC2435566 17317645

[80] L. V. Hedges , J. Gurevitch , and P. S. Curtis , “The meta‐analysis of Response Ratios in Experimental Ecology,” Ecology 80 (1999): 1150–1156.

[81] C. M. Pittelkow , X. Liang , B. A. Linquist , K. J. van Groenigen , et al., “Productivity Limits and Potentials of the Principles of Conservation Agriculture,” Nature 517 (2015): 365–368. 25337882 10.1038/nature13809

[82] D. Bates , M. Mächler , B. Bolker , and S. Walker , “Fitting Linear Mixed‐Effects Models Using lme4,” Journal of Statistical Software 67 (2015): 1–48.

[83] J. B. Johnson and K. S. Omland , “Model Selection in Ecology and Evolution,” Trends in Ecology & Evolution 19 (2004): 101–108. 16701236 10.1016/j.tree.2003.10.013

[84] J. Cohen and P. Cohen , Applied Multiple Regression/Correlation Analysis for the Behavioral Sciences (1983), 10.32614/CRAN.package.MuMIn(2010).

[85] M. Amiri , H. R. Pourghasemi , G. A. Ghanbarian , and S. F. Afzali , “Assessment of the Importance of Gully Erosion Effective Factors using Boruta Algorithm and its Spatial Modeling and Mapping using three Machine Learning Algorithms,” Geoderma 340 (2019): 55–69.

[86] M. Loreau and A. Hector , “Partitioning Selection and Complementarity in Biodiversity Experiments,” Nature 412 (2001): 72–76. 11452308 10.1038/35083573

[87] E. Paradis , et al., “ape: Analyses of Phylogenetics and Evolution,” 10.32614/CRAN.package.ape(2002). 14734327

[88] S. Ren , C. Terrer , J. Li , Y. Cao , S. Yang , and D. Liu , “Historical Impacts of Grazing on Carbon Stocks and Climate Mitigation Opportunities,” Nature Climate Change 14 (2024): 380–386.

[89] R Core Team R: A Language and Environment for Statistical Computing. R Foundation for Statistical Computing Vienna.

