# Supplementary material for: Optimal Grazing Exclusion Duration to Enhance Soil Carbon Sequestration in Degraded Grasslands
Source: Adv Sci (Weinh). 2026 Mar 12;13(29):e22212. doi: 10.1002/advs.202522212 (PMC13205864; doi:10.1002/advs.202522212)
Supplement: Supplementary file 1 — Supporting File: advs74783‐sup‐0001‐SuppMat.docx. [file ADVS-13-e22212-s001.docx]

**Supplementary Information**

**Supplementary Table 1**. List of 125 papers used in the meta-analysis.

| Data source | Reference number  (from main text reference list) |
| --- | --- |
| Shi XM, Li XG, Li CT, Zhao Y, Shang ZH, Ma QF. 2013. Grazing exclusion decreases soil organic carbon storage at an alpine grassland of the Qinghai-Tibetan Plateau. Ecological Engineering, 57, 183-187. | 1 |
| Wang CH, Han XG, Xing XR. 2010. Effects of grazing exclusion on soil net nitrogen mineralization and nitrogen availability in a temperate steppe in Northern China. Journal of Arid Environment, 74, 1287-1293. | 2 |
| Pei SF, Fu H, Wan CG. 2008. Changes in soil properties and vegetation following exclure and grazing in degraded Alxa desert steppe of Inner Mongolia, China. Agriculture, Ecosystems and Environment, 124, 33-39. | 3 |
| Li YQ, Zhou XH, Brandle JR, Zhang TH, Chen YP, Han JJ. 2012. Temporal process in improving carbon and nitrogen storage by grazing exclosure practice in a degraded land area of China's Horqin Sand Grassland. Agriculture, Ecosystems and Environment, 159, 55-61. | 4 |
| AO Yiming, JIAO Yan, XU Zhu. 2011. The changes of carbon and nitrogen storage of plant-soil system of enclosure years in typical steppe. Ecology and Environment Science. 20, 1403-1410. | 5 |
| Cao JJ. 2010. Effects of Grassland Management Changes on Soil Organic Carbon and Nitrogen Pools in the Qilian Mountains, China. Gansu Agricultural University. Master thesis (in Chinese with English abstract). | 6 |
| Chao ZG, Wang SP, Xu GP, Hu YG, Zhang ZH. 2008. Effect of Fencing on Community Structure and Distribution Patterns of Main Populations in Degraded *Kobresia humilis* Meadow. Acta Botanica Boreali-Occidentalia Sinica, 28, 2320-2326 (in Chinese with English abstract) | 7 |
| Chen YP, Li YQ, Zhao XY, Luo YQ, Shang W. 2010. Light Fraction and Total Organic Carbon and Nitrogen Stores in Desertified Sandy Grassland Soil as Affected by Grazing and Livestock Exclusion. Journal of Soil and Water Conservation, 24, 182-186 (in Chinese with English abstract) | 8 |
| Zhao S. 2011. Effects of grazing and fenced on soil microbial diversity in Stipa steppes of Hulunbeier, Inner Mongolia. Chinese Academy of Agricultural Sciences, Master thesis (in Chinese with English abstract). | 9 |
| Cui X, Wang Y, Niu H, Wu J, Wang S, Schnug E, Rogasik J, Fleckenstein J, Tang Y. 2005. Effect of long-term grazing on soil organic carbon content in semiarid steppes in Inner Mongolia. Ecological Research, 20, 519-527 | 10 |
| Yan YC, Tang HP, Chang RY, Liu L. 2008a. Study on the Difference of Vegetation and Soil in Typical Steppe Communities Under Different Fenced Time. Journal of Arid Land Resources and Environment, 22, 145-151 (in Chinese with English abstract) | 11 |
| Deng, L., Zhang, Z., & Shangguan, Z. 2014. Long-term fencing effects on plant diversity and soil properties in China. Soil and Tillage Research, 137, 7-15 | 12 |
| Dong, J. (2007). Study on the soil character and soil seed bank of fencing on the degenerative Typical steppe. Inner Mongolia Agricultural University, Master thesis (in Chinese with English abstract). | 13 |
| Fan Y, Hou X, Shi H, Shi, S. 2013. Effects of grazing and fencing on carbon and nitrogen reserves in plants and soils of alpine meadow in the three headwater resource regions. Russian Journal of Ecology, 44, 80-88 | 14 |
| Yan YC, Tang HP. 2008b. The restoration of degraded grassland and its contribution to carbon sequestration in the exclosure in Typical Steppe of Inner Mongolia, China. Progress in Natural Science, 18, 546-551 (in Chinese) | 15 |
| Gao Y, Zeng X, Schumann M, Chen H. 2011. Effectiveness of Exclosures on Restoration of Degraded Alpine Meadow in the Eastern Tibetan Plateau. Arid Land Research and Management, 25, 164-175 | 16 |
| He N, Zhang Y, Dai J, Han X, Baoyin T, Yu G. 2012. Land-use impact on soil carbon and nitrogen sequestration in typical steppe ecosystems, Inner Mongolia. Journal of Geographical Sciences, 22, 859-873 | 17 |
| He NP, Han XG, Yu GR. 2011. Carbon and nitrogen sequestration rate in long-term fenced grasslands in Inner Mongolia, China. Acta Ecologica Science, 31, 4270-4276 (in Chinese with English abstract) | 18 |
| Hu YK, Gao GG, Li KH, Gong YM, Yin W, Wang J, Chen AL. 2009. The Succession of Plant Communities in Alpine Grasslands in different Ages of Enclosing. Journal of Glaciology and Geocryology, 31, 1186-1194 (in Chinese with English abstract) | 19 |
| Jia, H.T. (2007). Ecological effects analysis of degraded grassland fencing in Xinjiang. Xinjiang Agricultural University, Master thesis (in Chinese with English abstract). | 20 |
| Li JB. 2008. Effects of Enclosing spring-autumn grassland on features of vegetation and soil at Zhaosu horse ranch. Xinjiang Agricultural University, Master thesis (in Chinese with English abstract) | 21 |
| Li Q, Yang J, Song B, et al. The impacts of different enclosure durations on degraded *Stipa grandis* grassland productivity and soil carbon and nitrogen storage. Chinese Journal of Ecology, 2014, 33(04), 896-901 | 22 |
| Luo XY, Jiao Y. 2012. Effects of Fencing Duration on CH_4_, Uptake in Typical Steppe. Acta Agrestia Sinica, 20, 1051-1058 (in Chinese with English abstract) | 23 |
| Pei SF. 2007. Effects of grazing and exclosure on soil and vegetation in Alsa desert steppe. Lanzhou University, Ph.D. thesis (in Chinese with English abstract) | 24 |
| Qiu L, Wei X, Zhang X, Cheng J. 2013. Ecosystem carbon and nitrogen accumulation after grazing exclusion in semiarid grassland. PLoS One, 8, e55433 | 25 |
| Sang Y, Jia C, Ruan WB, Ma CC, Gao YB. 2010. Effect of fencing on plant and nematode communities in the grassland in midand eastern Inner Mongolia, China. Ecology and Environmental Sciences, 19, 2332-2338 (in Chinese with English abstract) | 26 |
| Shan GL, Xu Z, Ning F, Jiao Y. 2009. Influence of seasonal exclosure on plant and soil characteristics in typical steppe. Acta Prataculturae Science, 18, 4-10(in Chinese with English abstract) | 27 |
| Shan GL, Xue SM, Chen G, Kuang CY, Liu ZL, Chu XH. 2012b. Influence of Seasonal Exclosure on Vegetation Restoration in Typical Steppe, Inner Mongolia. Acta Agrestia Sinica, 20, 812-818 (in Chinese with English abstract) | 28 |
| Shi XM, Li XG, Li CT, Zhao Y, Shang ZH, Ma Q. 2013. Grazing exclusion decreases soil organic C storage at an alpine grassland of the Qinghai–Tibetan Plateau. Ecological Engineering, 57, 183-187 | 29 |
| Su YZ, Zhao HL. 2003. Influences of Grazing and Exclosure on Carbon Sequestration in Degraded Sandy Grassland, Inner Mongolia, North China. Environmental Science, 24, 23-28 (in Chinese with English abstract) | 30 |
| Su YZ, Li YL, Cui HY, Zhao WZ. 2005. Influences of continuous grazing and livestock exclusion on soil properties in a degraded sandy grassland, Inner Mongolia, northern China. Catena, 59, 267-278. | 31 |
| Sun ZJ, An SZ, Duan JJ. 2009. Effect of Enclosure on Vegetation and Soil Nutrient of Sagebrush Desert Grassland in Xinjiang. Arid Zone Research, 26, 877-882 (in Chinese with English abstract) | 32 |
| Wen HY, Shang ZH, Fu H. 2005. Effects of years for reclamation and enclosure years on soil properties of degraded sandy grassland. Acta Prataculturae Science, 14, 31-37 (in Chinese with English abstract) | 33 |
| Wu GL, Liu ZH, Zhang L, Chen JM, Hu TM. 2010. Long-term fencing improved soil properties and soil organic carbon storage in an alpine swamp meadow of western China. Plant and Soil, 332, 331-337 | 34 |
| Wu JB, Bao XY, Li J, Zhao NX, Gao YB. 2010. Influence of Fencing Duration on Community and Population of Stipa grandis in a Typical Steppe. Acta Agrestia Sinica, 18, 490-495 (in Chinese with English abstract) | 35 |
| Xue B. 2008. The influence of enclosing on soil and vegetation on the degradation grassland in Duolun Inner Mongolia Agricultural University. Master thesis (in Chinese with English abstract). | 36 |
| Wei D, Ri X, Wang Y, Wang Y, Liu Y, Yao T. 2012. Responses of CO_2_, CH_4_ and N_2_O fluxes to livestock enclosure in an alpine steppe on the Tibetan Plateau, China. Plant and Soil, 359, 45-55 | 37 |
| Wang CY, Zhang JJ, LV YL, Wang L, He NP. 2014. Effects of long-term grazing exclusion on soil organic carbon fractions in the grasslands of Inner Mongolia. Acta Prataculturae Sinica, 23, 31-3 | 38 |
| Yu XJ, Jing YY, Duan CH, Xu CL, Yang HL. 2015. Influence of enclosure and grazing intensity on alpine meadow vegetation and soil characteristics in the Eastern Qilian Mountains. Agricultural Research in the Arid Areas, 33, 252-258 (in Chinese) | 39 |
| Zhou T, Gao J, Wang J, et al. Effects of 7-Year Enclosure on Plant Community Structure and Soil Physico-Chemical Properties at the Southeastern Margin of the Tibetan Plateau. Acta Prataculturae Sinica 2018,27(12), 1-11 | 40 |
| Li Q, Song YT, Zhou DW, Wang ML, Chen XY. 2014. Effects of fencing and grazing on soil carbon, nitrogen, phosporus storage in degraded alkali-saline grassland. Pratacultural Science. 31, 1811-1819 (in Chinese) | 41 |
| Sun DC, Li YL, Zhao XY, Zuo XA, Mao W. 2015. Effects of enclosure and grazing on carbon and water fluxes of sandy grassland. Chinese Journal of Plant Ecology, 39, 565-576 (in Chinese) | 42 |
| Sun DC, Li YL, Zhao XY, Luo YY, Bi JD. 2016. Effects of grazing and enclosure on net ecosystem carbon exchange in the Horqin sandy grassland. Chinese Desert, 36, 93-102 (in Chinese) | 43 |
| Li Y, Yan ZY, Guo D, Wang HX, Su SL, Li XD, Fu H. 2015. Effects of fencing ang grazing on vegetation and soil physical and chemical properties in an alpine meadow in the Qinghai Lake Basin. Acta Prataculturae Sinica, 24, 33-39 (in Chinese) | 44 |
| Si GC, Yuan YL, Wang J, Wang GP, Lei TZ, Zhang GX. 2015. Effects of fencing on microbial communities and soil enzyme activities in Damxung alpine grassland. Pratacultural Science. 32, 1-10 (in Chinese) | 45 |
| Zhao CZ, LongRJ. Rehabilitation process of degraded *Melica Przewalskyi* grassland in the upper reaches of Shiyang River. Journal of Mountain Science, 26, 286-292 (in Chinese) | 46 |
| Xie J, Fan YM, Wu HQ, Guan GY, Chai DP, He J. 2014. Effects of enclosure on soil quality in Mountain Desert grassland. Xinjiang Agricultural Sciences, 15, 1699-1705. (in Chinese) | 47 |
| He GY, Sun HZ, Shi XM, Qi W, Du GZ. 2015. Soil properties of Tibetan plateau alpine wetland affected by grazing and season. Acta Prataculture Sinica, 24, 12-20 (in Chinese) | 48 |
| Zhang FW, Li YN, Wang SP, Zhao XQ. 2009. Response of soil organic matter, total nitrogen and total phosphor to different land use patterns in alpine meadow of Qinghai-Tibetan Plateau. Chinese Journal of Agrometeorology, 30, 323-326 (in Chinese) | 49 |
| Tian JQ, Zhou ZY, Bao B, Sun JX. 2008. Variations of soil particle size distribution with land -use types and influences on soil organic carbon and Nitrogen. Journal of plant ecology, 32, 601-610 (in Chinese) | 50 |
| Shen Y, Ma HB, Xie YZ, Du DM, Zhao F. 2012. Response to different management modes of soil physical and chemical prosperities in typical steppe, Ningxia. Journal of soil and water conservation, 26, 84-89 (in Chinese) | 51 |
| Hao WF, Liang ZS, Cheng GG, Tang L. Study of the different succession stage community dynamic and the evolution of soil characteristics of the old-field in loess hills gully. Chinese Agricultural Science Bulletin, 21, 226-231 (in Chinese) | 52 |
| Shen XK, Liu MH, Zhang YK, Zhang X, Fu H. 2014. Carbon exchange characteristics of fenced and natural grazed grassland in Loess Plateau. Acta Bot. Boreal. 34, 1869-1877 (in Chinese) | 53 |
| Dai JZ, Wei ZJ, He NP, Wang RM, Wen XH, Zhang YH, Zhao XN, Yu GR. 2012. Effect of grazing exclosure on the priming effect and temperature sensitivity of soil C mineralization in Leymus chinensis graslands, Inner Mongolia, China. Chinese Journal of Plant Ecology, 36, 1226-1236 (in Chinese) | 54 |
| Xue Y, Zong N, He N, Tian J, Zhang Y. Influence of long-term enclosure and free grazing on soil microbial community structure and carbon metabolic diversity of alpine meadow. 2018, 29 (08), 2705-2712 | 55 |
| Guan GY, Fan YM, Wu HQ, Gui F, Li KN, Li F. 2014. Effects of fencing on soil active organic carbon and carbon pool management index in mountain meadow steppe. Pratacultural Science, 31, 1618-1622 (in Chinese) | 56 |
| Zhao S, Zhang JN, Lai X, Yang DL, Zhao JN, Li G, Zou YK. 2011. Analysis of microbial biomass C, N and soil microbial community structure of Stipa Steppes Using PLFA at grazing and fenced In Inner Mongolia, China. Journal of Agro-Environment Science, 30, 1126-1134 (in Chinese) | 57 |
| Sun DC, Li YL, Zhao XY, Mao W, Yue XF. 2015. Effects of grazing and enclosure on soil respiration rate in the Horqin Sandy Grassland. Journal of Desert Research, 35, 1620-1627 (in Chinese) | 58 |
| Zheng JG, He MZ, Su Y, Xu JG, Li XR. 2011. Effects of grazing and enclosure on desert grassland ecosystem. Journal of Henan Agricultural Sciences, 40, 80-83 (in Chinese) | 59 |
| Yang XG, Song NP, Li XB, Liu BR. 2012. Effects of short-term fencing on organic carbon fractions and physical stability of sand sierozem in desert steppe of Northwest China. Chinese Journal of Applied Ecology, 23, 3325-3330 (in Chinese) | 60 |
| Shan GL, Chu XH, Tian QS, Ma YB, Li LH, Chen G. 2012c.Research on the dynamic changes of soil properties of typical steppe in the restoring process. Acta Prataculturae Sinica, 21, 1-9 (in Chinese) | 61 |
| Shan GL, Chen G, Ning F, Ma YB, Chu XH. 2012d. Dynamics of soil microorganism and enzyme activity in typical steppe of restoration succession process. Acta Prataculturae Sinica, 20, 292-297. (in Chinese) | 62 |
| Zhao CX, zheng DW, He WQ, Pan ZH, Hu YG, Fan XR. 2006. Study on plant community characters and soil properties of Artemisia firigida grasslands after different enclosing times. Pratacultural Science, 23, 89-92 (in Chinese) | 63 |
| Fang F, Tang HP, Li BY. 2013. Effects of land use type on soil organic carbon and its fractions. Ecology and Environment, 22, 1774-1779. (in Chinese) | 64 |
| Li W, CaoWX, Liu HD, Li XL, Xu CL, Shi SL, Feng J, Zhou CM. Analysis of soil respiration under different grazing management patterns in the alpine meadow-steppe of the Qinghai-Tibet Plateau. Acta Prataculturae Sinica, 24, 22-32 (in Chinese) | 65 |
| Fu H, Chen Ym, Wang YR, Wan CG. 2004. Organic carbon content in major grassland types in Alex, Innner Mongolia. Acta Ecologica Sinica, 24, 469-476 (in Chinese) | 66 |
| Jing ZB, Cheng JM, Su J, Bai Y, Jin JW. 2014. Changes in plant community composition and soil properties under 3-decade grazing exclusion in semiarid grassland. Ecological Engineering, 64, 171-178 | 67 |
| Luan JW, Cui LJ, Xiang CH, Wu JH, Song HT, Ma QF, Hu ZD. 2014. Different grazing removal exclosures effects on soil C stocks among alpine ecosystems in east Qinghai-Tibet Plateau. Ecological Engineering, 64, 262-268 | 68 |
| Wu X, Li ZS, Fu BJ, Zhou WM, Liu HF, Liu GH. 2014. Restyoration of ecosystem carbon and nitrogen storage and microbial biomass after grazing exclusion in semi-arid grasslands of Inner Mongolia. Ecological Engineering, 73, 395-403 | 69 |
| Ma WM, Ding KY, Li ZW. 2016. Comparison of soil carbon and nitrogen stocks at grazing-excluded and yak grazed alpine meadow sites in QINGHAI-Tibetan Plateau, China. Ecological Engineering, 87, 203-211 | 70 |
| Zhu GY, Deng L, Zhang XB, Shangguan ZP. 2016. Effects of grazing exclusion on plant community and soil physicochemical properties in a desert steppe on the Loess Plateau, China. Ecological Engineering, 90, 372-381 | 71 |
| Wang KB, Deng L, Ren ZP, Li JP, Shangguan ZP. 2016. Grazing exclusion significantly improves grassland ecosystem C and N pools in a desert steppe of Northwest China. Catena, 137, 441-448 | 72 |
| Wu X, LI Zs, Fu BJ, Lu F, Wang DB, Liu HF, Liu GH. 2014. Effects of grazing exclusion on soil carbon and nitrogen storage in semi-arid grassland in Inner Mongolia. Chinese Geographical Science, 24, 479-487 | 73 |
| Wen HY, Niu DC, Fu H, Kang J. 2013. Experimental investigation on soil carbon, nitrogen, and their components under grazing and livestock exclusion in steppe and desert steppe grasslands, Northwestern China. Environ Earth Sci, 70, 3131-3141 | 74 |
| Chen YP, Li YQ, Zhao XY, Awada T, Shang W, Han JJ. 2012. Effects of grazing exclusion on Soil Properties and on ecosystem carbon and nitrogen storage in a sandy rangeland of Inner Mongolia, Northern China. Environmental Management, 50, 622-632 | 75 |
| Li YQ, Zhao HL, Zhao XY, Zhang TH, Li YL, Cui JY. 2011. Effects of grazing and livestock exclusion on soil physical and chemical properties in decertified sandy grassland, inner Mongolia, northern China. Environment Earth Science, 63, 771-783 | 76 |
| Li Q, Zhou DW, Jin YH, Wang ML, Song YT, Li GD. 2014. Effects of fencing on vegetation and soil restoration in a degraded alkaline grassland in northeast China. Journal of Arid Land, 6, 478-487 | 77 |
| Zhao L, Wu W, Xu X, Yu Y. 2014. Soil organic matter dynamics under different land use in grasslands in Inner Mongolia (Northern China). Biogeosciences, 11, 5103-5113 | 78 |
| Li YQ, Zhao XY, Chen YP, Luo YQ, Wang SK. 2012. Effects of grazing exclusion on carbon sequestration and the associated vegetation and soil characteristics at a semi-arid decertified sandy site in Inner Mongolia, northern China. Can. J. Soil.Sci. 92, 807-819 | 79 |
| Wang D, Wu GL, Zhu YJ, Shi ZH. 2014. Grazing exclusion effects on above-and below-ground C and N pools of typical grassland on the Loess Plateau (China). Catena, 123, 113-120 | 80 |
| Wu L, Wang HY, Han X. 2008. Storage and dynamics of carbon and nitrogen in soil after grazing exclusion in *Leymus chinensis* grasslands of Northern China. J, Environ. Qual., 37, 663-668 | 81 |
| Qiu L, Wei X, Zhang X, Cheng J. 2013. Ecosystem carbon and nitrogen accumulation after grazing exclusion in semiarid grassland. PLoS One, 8, e55433 | 82 |
| Wen D, He NP, Zhang JJ. 2016. Dynamics of soil organic carbon and aggregate stability with grazing exclusion in the Inner Mongolian Grasslands. Plos One, 11, e0146757 | 83 |
| Zhang Q, Deng J, Mao J, Duo Y, Cheng J, Guo L. Impacts of Different Enclosure Ages on Soil and Microbial Carbon and Nitrogen Contents in Semi-arid Grasslands, 2021, 40, 29-34 | 84 |
| Dong Y, Sun Z, An S, Yang J. Effects of grazing exclusion on soil nutrition in moderate degraded desert grassland of *Seriphidium transiliense*. Pratacultural Science, 2016, 10(8), 1460-1468 | 85 |
| Jing J, Zhang M, Gao Y. Effects of enclosure on soil microbial carbon utilization in an alpine steppe. Ecological Science, 2021, 40, 25-32 | 86 |
| Zhang J, Li Y, Zhao X, Zhang T, She Q, Liu M, Wei S. Effects of Exclosure on Soil Physicochemical Properties and Carbon Sequestration Potential Recovery of Desertified Grassland. Journal of Desert Research, 2017, 37, 491-499 | 87 |
| Yang Y, Liu A, Li H, Chen H, Song X, Wang B, Luo D, Wang M. Effects of Fertilization on Vegetation Community Characteristics and Soil Properties of a Typical Steppe in Inner Mongolia. Acta Prataculturae Sinica, 2016, 25, 21-29 | 88 |
| Gao Y, Liao L, Wang J, Wan Q, Liu G, Zhang C. Effects of Grazing Exclusion on the Fractional Characteristics of Soil Particle Size in Semi-arid Grassland and on the Loose Plateau. Journal of Soil and Water Conservation, 2021, 35, 310-326 | 89 |
| Zhang S. Effects of grazing exclusion on plant community composition and carbon storage of alpine meadow in Qinghai-Tibet Plateau. Lanzhou University. Master thesis (in Chinese with English abstract) | 90 |
| Chai Q. Effects of different managements on community structure and ecological functions in a typical steppe. Northwest A&F University. Master thesis (in Chinese with English abstract) | 91 |
| Jiang S, Zhang W, Mu H, et al. Effects of Management on Plant and Soil Physicochemical Characteristics of Typical Steppe. Chinese Journal of Grassland, 2022, 44, 83-89 | 92 |
| Han C, Yang Y, Liu B, et al. The Impact of Exclosure Age on Soil Organic Carbon, Total Nitrogen, Total Phosphorus, and Microbial Biomass Carbon and Nitrogen in Desert Grasslands. Jiangsu Agricultural Sciences, 2017, 45, 260-263 | 93 |
| Tenzin Tarchen, Baima Gaweng, Duoji Dunzhu, Laba. Effects of livestock exclusion duration years on plant and soil properties in a Tibetan alpine meadow. Pratacultural Science, 2018, 35(1), 10-17 | 94 |
| Yu G, Li K, Zhou J, et al. Effects of long-term enclosure on soil aggregate stability and erodibility in Bayinbuluk alpine grassland. Arid zone research. 2022, 39, 1842-1851 | 95 |
| Suo L. Effects of enclosure on vegetation-soil features of the *leymus chinensis* grassland: emphasizing the changes in soil organic carbon fractions. Inner Mongolia University. Master thesis (in Chinese with English abstract) | 96 |
| Gao F, Wang B, Shi Y X, Zhang G X, Wang J, Si G C, Han C H, Yuan Y L, Hu A. The response of alpine grasslands ecosystem in the north Tibet to short-term enclosure. Acta Ecologica Sinica, 2017, 37(13), 4366-4374 | 97 |
| Zhang H. Responses of Soil Carbon Stocks in the Grasslands to Enclosures in the Typical Steppe on the Loess Plateau, China. Ningxia University. Master thesis (in Chinese with English abstract) | 98 |
| Zhang X, Meng Z, Yang Z. Soil Quality Assessment under Different Exclusion Measures in Desert Steppe in Xilamuren, Inner Mongolia. Chinese journal of soil science. 2018, 49, 788-793. | 99 |
| Xu D, Xu X, Wang G, et al. Variations in soil organic carbon content and distribution during natural restoration succession on the desert steppe in Ningxia. Acta Prataculturae Sinica. 2017, 26, 35-42 | 100 |
| Zhou Yao, Ma Hong-Bin, Jia Xi-Yang, et al. Effects of different restoration measures on storage of soil organic carbon and nitrogen in typical steppe of the Loess Hilly Area in Ningxia. Acta Prataculturae Sinica, 2017, 26(12), 236-242 | 101 |
| Du, Chenjun, Jie Jing, Yuan Shen, Haixiu Liu, and Yongheng Gao. Short-term Grazing Exclusion Improved Topsoil Conditions and Plant Characteristics in Degraded Alpine Grasslands. Ecological Indicators, 108.C (2020), 105680 | 102 |
| Wang, K. et al. (2016) Grazing exclusion significantly improves grassland ecosystem C and N pools in a desert steppe of Northwest China. Catena (Giessen), 137, 441–448 | 103 |
| Wen Li, Wenxia Cao,Jinlan Wang, Xiaolong Li, Changlin Xu, Shangli Shi. Effects of grazing regime on vegetation structure, productivity, soil quality, carbon and nitrogen storage of alpine meadow on the Qinghai-Tibetan Plateau.Ecological Engineering, 2017(98), 123–133 | 104 |
| Yuan, Z., Epstein, H., & Li, G. (2020). Grazing exclusion did not affect soil properties in alpine meadows in the Tibetan permafrost region. Ecological Engineering, 147, 105657 | 105 |
| Zhao, J., Sun, F., & Tian, L. (2019). Altitudinal pattern of grazing exclusion effects on vegetation characteristics and soil properties in alpine grasslands on the central Tibetan Plateau. Journal of Soils and Sediments, 19(2), 750-761 | 106 |
| Liu, J., Wu, J., Su, H., Gao, Z., & Wu, Z. (2017). Effects of grazing exclusion in Xilin Gol grassland differ between regions. Ecological Engineering, 99, 271-281 | 107 |
| Cong, S., Zhou, D., Li, Q., & Huang, Y. (2021). Effects of Fencing on Vegetation and Soil Nutrients of the Temperate Steppe Grasslands in Inner Mongolia. Agronomy (Basel), 11(8), 1546 | 108 |
| Hu, J., Zhou, D., Li, Q., & Wang, Q. (2021). Effects of Long-Term Enclosing on Vertical Distributions of Soil Physical Properties and Nutrient Stocks in Grassland of Inner Mongolia. Agronomy (Basel), 11(9), 1832 | 109 |
| Li, J., Shangguan, Z., & Deng, L. (2022). Free particulate organic carbon plays critical roles in carbon accumulations during grassland succession since grazing exclusion. Soil & Tillage Research, 220, 105380 | 110 |
| Zeng, Q., Liu, Y., Xiao, L., & Huang, Y. (2017). How Fencing Affects the Soil Quality and Plant Biomass in the Grassland of the Loess Plateau. International Journal of Environmental Research and Public Health, 14(10), 1117 | 111 |
| Zhang H, Li J, Wang Y, et al. Effects of Enclosures and Grazing on Soil Stoichiometry Characteristics of Natural Grassland on the Loess Plateau. Journal of Soil and Water Conservation, 2020, 34, 251-258 | 112 |
| G.H. Ren, C.X. Wang, K.H. Dong, H.S. Zhu, Y.C. Wang, X. Zhao, Effects of grazing exclusion on soil-vegetation relationships in a semiarid grassland on the Loess Plateau, China. Land Degradation & Development, 29(11), (2018), 4071-4079 | 113 |
| Hao F, Zhang J, Xing P, et al. Effects of Enclosures on the Eco-stoichiometric Characteristics of Carbon, Nitrogen, and Phosphorus in *Leymus secalinus* Grassland on the North Shaanxi and Their Relationship with Plant Diversity. Acta Agrestia Sincia. 2019, 27, 644-650 | 114 |
| Jing Jia-qiang, SA Ren-qi-li-mo-ge, Qin Jie, et al. Effects of different land-use patterns on soil active organic carbon in *Stipa baicalensis* steppe in Inner Mongolia. Acta Prataculturae Sinica, 2022, 31(1), 47−56 | 115 |
| Yu S, Tao LB, Xu D, et al. Effects of enclosure on the soil organic carbon and its active components in desert steppe grassland. Acta Prataculturae Sinica, 2019, 28(2), 190-196 | 116 |
| Sarenqilimog, Jing J, Qin J, et al. Ecological Stoichiometric Characteristics of Plants and Soil in *Stipa baicalensis* Grassland under Different Utilization Modes. Chinese Journal of Grassland. 2022, 44, 20-29 | 117 |
| Zhang P, Ma J, Cheng J, et al. Effects of Enclosures on Vegetation Characteristics and Soil Physical and Chemical Properties of Temperate Grassland. Chinese Journal of Grassland. 2021, 43, 41-50 | 118 |
| Zhao L, Zhang X, Xiong B, et al. Influence of Fencing and Grazing on the Soil and Standing Vegetation Changes in HorQin Sandy Grassland. Ecology and Environmental Sciences. 2017, 26, 971-977 | 119 |
| Wang N. Effects of Different Years of Enclosures on Soil and Vegetation of the Zhenlanqi Grassland. Beijing Forestry University. Master thesis (in Chinese with English abstract). | 120 |
| Li G, Zhao P, Shao W, et al. Studies on the soil physical and chemical properties and enzyme activities of two fenced plant communities in desert steppe grassland. Acta Prataculturae Sincia, 2019, 28, 49-59 | 121 |
| Luo D, Wang M, Zheng S, et al. Effects of enclosure on soil microbial quantity and enzyme activity in desert steppe. Ecology and Environmental Sciences, 2016, 25, 760-767 | 122 |
| Li J, Dong Q, Tursunay R, et al. Effects of enclosure on soil physical and chemical quality and enzymatic activity in grassland of Yili valley in spring autumn. Chinese Journal of Grassland, 2014, 36, 84-89 | 123 |
| Mi Q, Wang Y, Qin X, et al. Response of Soil Enzyme Stoichiometry to Different Enclosures Durations in Alpine Meadow, Tibetan Plateau. Acta Agrestia Sincia, 2021, 29, 33-41 | 124 |
| Ao Y. Study on soil ecological stoichiometry of enclosing life in typical steppe. Inner Mongolia Normal University. Master thesis (in Chinese with English abstract) | 125 |


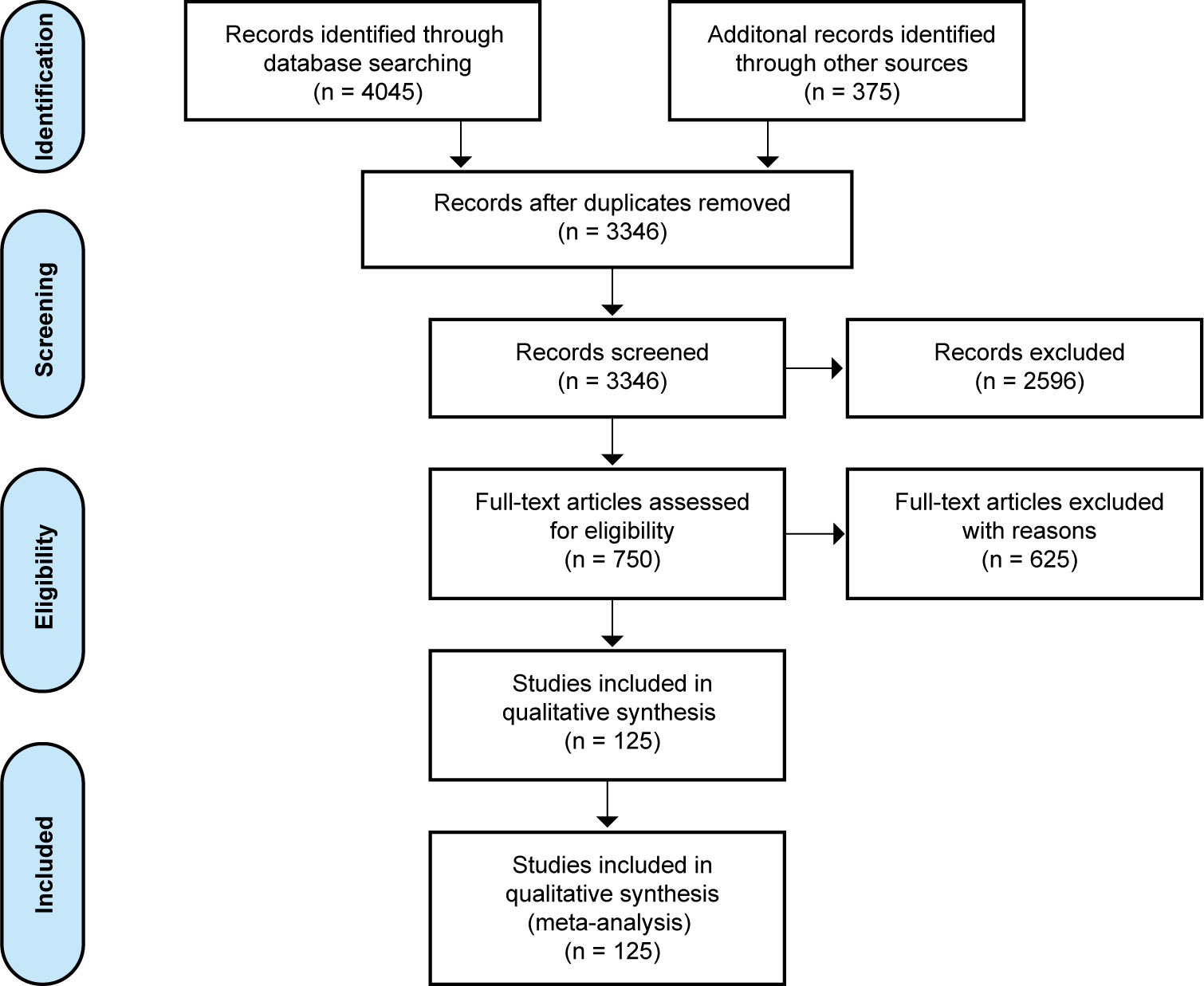


**Supplementary Figure 1**. PRISMA diagram showing the process of identifying eligible studies to be included in this meta-analysis.


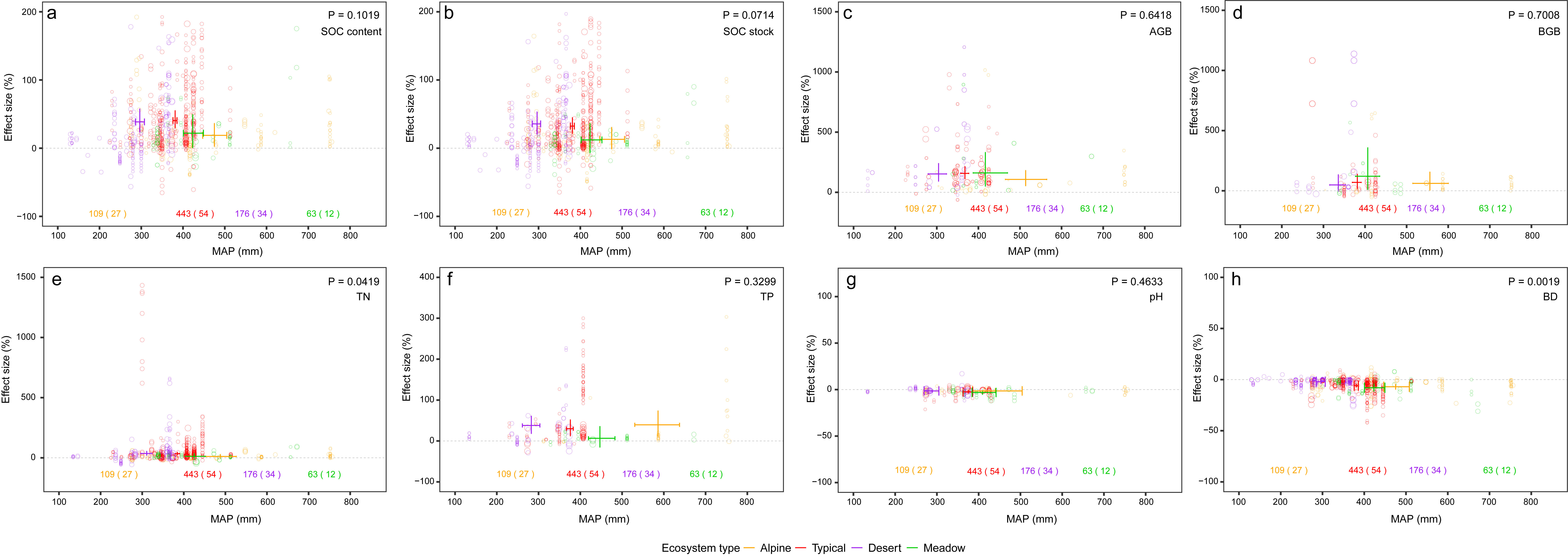


**Supplementary Figure 2**. Comparison of environmental attributes in different mean annual precipitation (MAP) areas among the four grassland types. Individual observations with point sizes corresponding to their weights (Wr, see Methods) are plotted by grassland type. a Soil carbon content. b Soil carbon storage. c Aboveground biomass. d Belowground biomass. e Total nitrogen. f Total phosphorus. g Soil pH. h Bulk density. Means and vertical and horizontal error bars represent means and 95% bootstrapped confidence intervals for the effect size (%) of mean annual precipitation. For each grassland type, the number of observations is shown without parentheses with the number of studies in parentheses. *P* value, derived from the linear mixed model with the grassland type as the fixed effect and the study as the random effect, represents the significance of the difference in the effect sizes among grassland types. The figure shows that despite significant differences in the mean annual precipitation among grassland types (as indicated by non-overlapping confidence intervals), only the effect sizes of soil bulk density and total nitrogen showed significant differences between the studied environmental attributes. The four grassland types considered were Alpine, Desert, Meadow, and Typical.


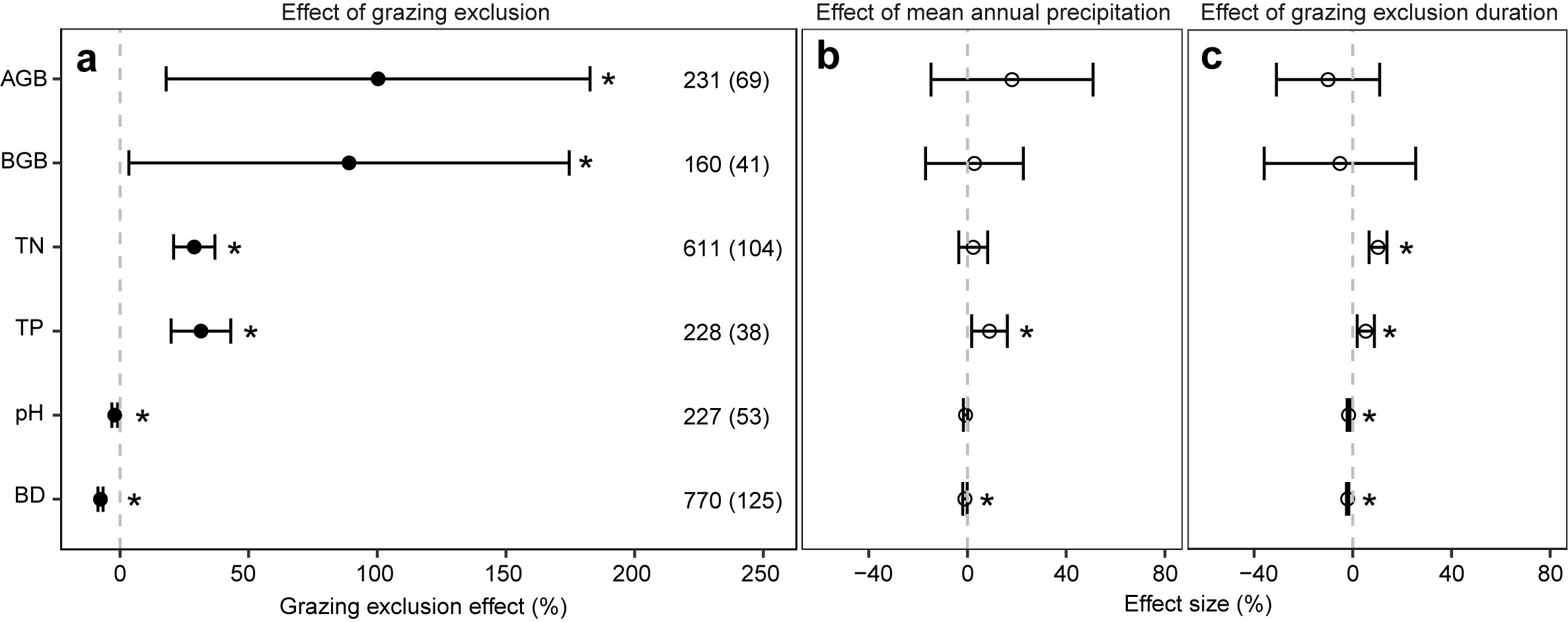


**Supplementary Figure 3**. The effects of grazing exclusion on environmental attributes in relation to mean annual precipitation and grazing exclusion duration. **a** The grazing exclusion. **b** The mean annual precipitation. **c** Grazing exclusion duration (years). The effects represent the estimated coefficients of the mean annual precipitation and grazing exclusion duration. Values (estimated *β_1_* and *β_2_* in Equation (3), respectively, see Methods) are mean ± 95% confidence intervals. AGB: aboveground biomass, BGB: belowground biomass, TN: total nitrogen, TP: total phosphorus, pH: soil pH, BD: bulk density. The number of observations in **a** is shown without parentheses above each attribute together with the number of studies in parentheses. Asterisks indicate significant difference or effect size at *P* < 0.05. Source data are provided as a Source Data file.


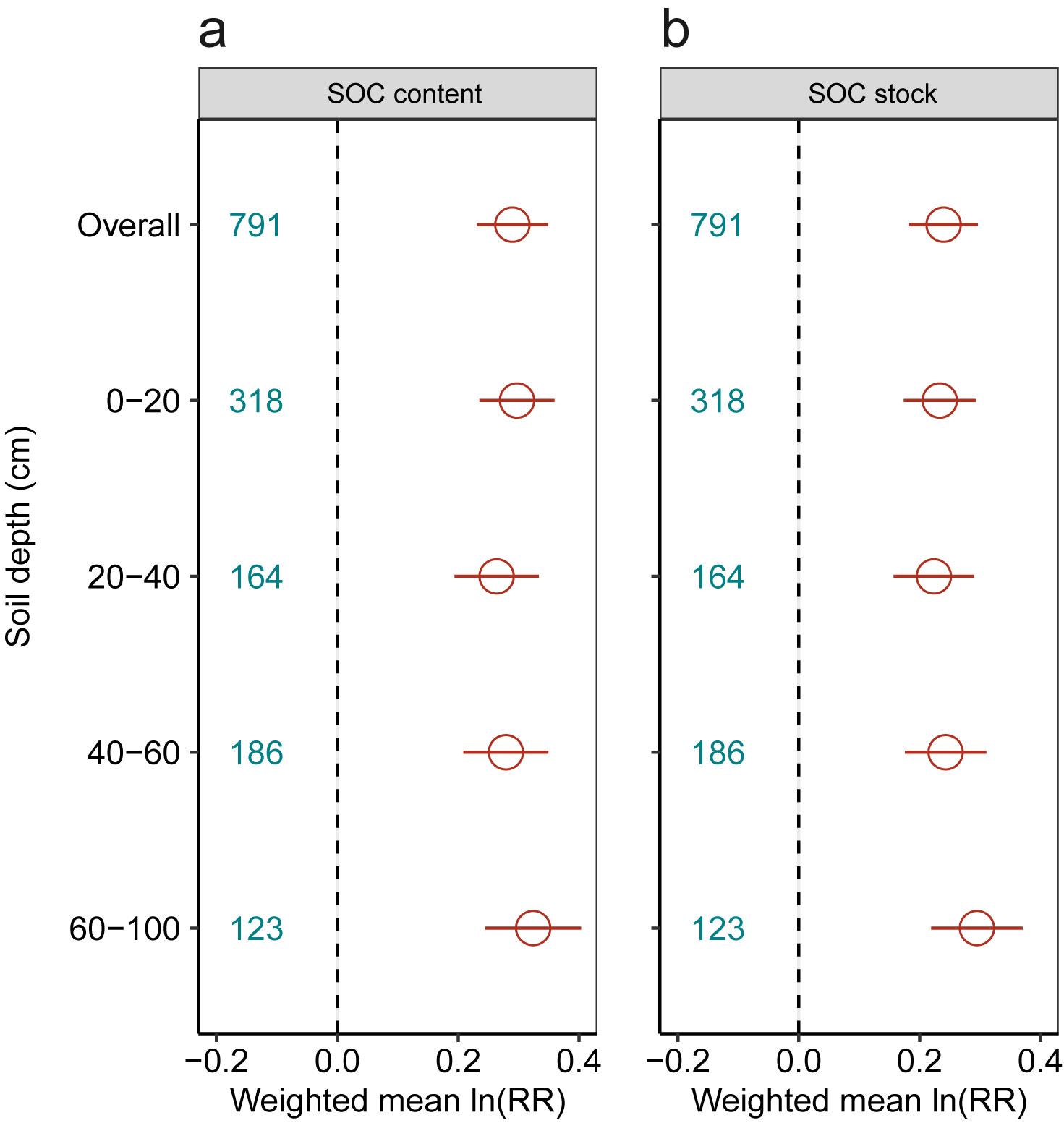


**Supplementary Figure 4**. The effects of grazing exclusion on soil organic carbon (SOC) content and SOC stock at different soil depths. The effects represent the estimated coefficients of the grazing exclusion. Values are mean ± 95% confidence intervals. The green number of observations in **a** and **b** is shown at left of each soil depth. Source data are provided as a Source Data file.


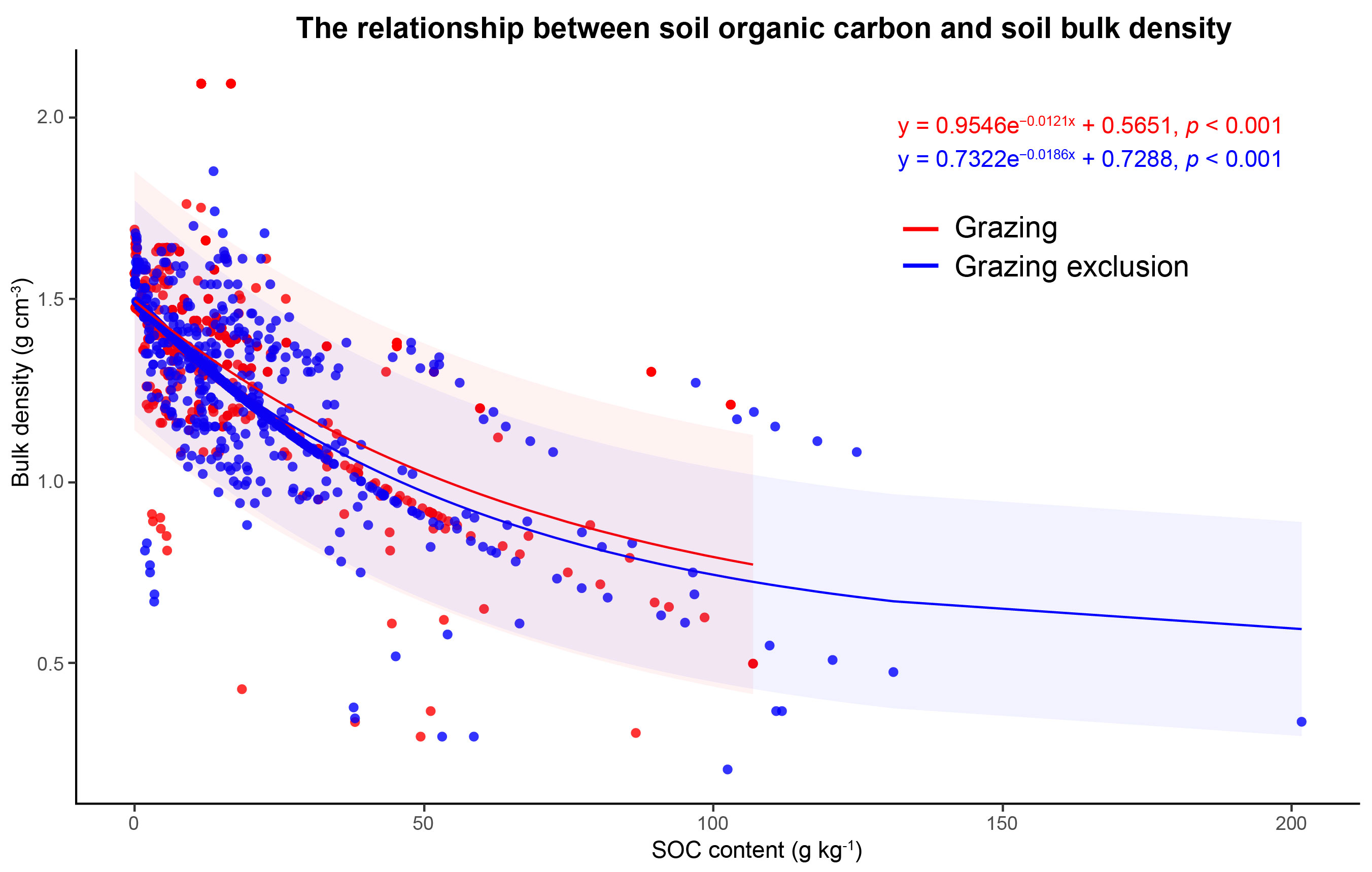


**Supplementary Figure 5**. Relationships between soil carbon content and bulk density across grazing and grazing exclusion experiments (both *P* < 0.001). Nonlinear fits depict exponential models with shaded areas representing 95% confidence interval. *P* values for two-tailed tests

**Supplementary Table 2**. The Akaike Information Criterion (AIC) values for the full model (Equation (3) in Methods; AIC1) and the most parsimonious model (AIC2), and the effects (*P* values) of the intercept (testing whether it differs from zero), the mean annual precipitation (P), grazing exclusion duration (T), and grassland type (L) of the most parsimonious models. For all environmental attributes except aboveground biomass and belowground biomass, grassland type-associated terms (Equation (3) in Methods) were excluded in the most parsimonious models. The corresponding coefficients and their bootstrapped 95% confidence intervals are presented in Fig. 3. *P* values are in bold when < 0.05.

| Attribute | Full model | |  | The most parsimonious model | | | | | | | | | | | | | |
| --- | --- | --- | --- | --- | --- | --- | --- | --- | --- | --- | --- | --- | --- | --- | --- | --- | --- |
|  | AIC1 |  |  | AIC2 |  | Intercept |  | Log(P) |  | Log(T) |  | Log(P) × Log(T) |  | L | Log(P) × L |  | Log(T) × L |
| SOC content | 373.818699 |  |  | 358.4808 |  | **<0.001** |  | **<0.001** |  | **<0.001** |  | **0.006** |  |  |  |  |  |
| SOC stock | 318.502462 |  |  | 303.8944 |  | **<0.001** |  | **0.002** |  | **<0.001** |  | **0.027** |  |  |  |  |  |
| Total nitrogen | 451.07962 |  |  | 427.6019 |  | **<0.001** |  | 0.468 |  | **<0.001** |  | 0.547 |  |  |  |  |  |
| Total phosphorus | 6.396269 |  |  | -26.6256 |  | **<0.001** |  | **0.016** |  | **0.003** |  | 0.059 |  |  |  |  |  |
| Soil pH | -824.504431 |  |  | -898.8636 |  | **<0.001** |  | 0.079 |  | **<0.001** |  | **0.003** |  |  |  |  |  |
| Bulk density | -2248.829892 |  |  | -2302.5337 |  | **<0.001** |  | **0.026** |  | **<0.001** |  | **0.002** |  |  |  |  |  |
| Aboveground biomass | 290.891397 |  |  | 290.5158 |  | **0.008** |  | 0.390 |  | 0.270 |  | **0.008** |  | Alpine | / |  | / |
|  |  |  |  |  |  |  |  |  |  |  |  |  |  | Desert | 0.420 |  | **<0.001** |
|  |  |  |  |  |  |  |  |  |  |  |  |  |  | Meadow | **<0.001** |  | **<0.001** |
|  |  |  |  |  |  |  |  |  |  |  |  |  |  | Typical | 0.821 |  | 0.123 |
| Belowground biomass | 280.984272 |  |  | 279.8965 |  | **0.041** |  | 0.931 |  | 0.526 |  | 0.089 |  | Alpine | / |  | / |
|  |  |  |  |  |  |  |  |  |  |  |  |  |  | Desert |  |  | **<0.001** |
|  |  |  |  |  |  |  |  |  |  |  |  |  |  | Meadow |  |  | 0.967 |
|  |  |  |  |  |  |  |  |  |  |  |  |  |  | Typical |  |  | 0.181 |

**Supplementary Table 3**. Akaike Information Criterion (AIC) values of four alternative scenarios (I, II, III, and IV) with the linear and log-linear mean annual precipitation (R), and grazing exclusion duration (A).

| Attribute |  | Ⅰ |  |  | Ⅱ |  |  | Ⅲ |  |  | Ⅳ |  |
| --- | --- | --- | --- | --- | --- | --- | --- | --- | --- | --- | --- | --- |
|  |  | Terms | AIC |  | Terms | AIC |  | Terms | AIC |  | Terms | AIC |
| SOC content |  | R + A + R × A | 333.23 |  | ln(R) + A + ln(R) × A | 334.41 |  | ln(R) + ln(A) + ln(R) × ln(A) | 358.48 |  | R + ln(A) + R × ln(A) | 359.61 |
| SOC stock |  | R + A + R × A | 272.27 |  | ln(R) + A + ln(R) × A | 272.93 |  | ln(R) + ln(A) + ln(R) × ln(A) | 303.89 |  | R + ln(A) + R × ln(A) | 305.15 |
| Total nitrogen |  | R + A + R × A | 414.63 |  | ln(R) + A + ln(R) × A | 414.43 |  | ln(R) + ln(A) + ln(R) × ln(A) | 427.60 |  | R + ln(A) + R × ln(A) | 427.75 |
| Total phosphorus |  | R + A + R × A | -31.57 |  | ln(R) + A + ln(R) × A | -29.37 |  | ln(R) + ln(A) + ln(R) × ln(A) | -26.63 |  | R + ln(A) + R × ln(A) | -27.44 |
| Soil pH |  | R + A + R × A | -894.95 |  | ln(R) + A + ln(R) × A | -893.35 |  | ln(R) + ln(A) + ln(R) × ln(A) | -898.86 |  | R + ln(A) + R × ln(A) | -900.98 |
| Bulk density |  | R + A + R × A | -2277.7 |  | ln(R) + A + ln(R) × A + | -2275.9 |  | ln(R) + ln(A) + ln(R) × ln(A) + | -2302.5 |  | R + ln(A) + R × ln(A) | -2303.9 |
| Aboveground biomass |  | R + A + R × A | 291.39 |  | ln(R) + A + ln(R) × A | 293.48 |  | ln(R) + ln(A) + ln(R) × ln(A) | 290.52 |  | R + ln(A) + R × ln(A) | 287.04 |
| Belowground biomass |  | R + A + R × A | 290.88 |  | ln(R) + A + ln(R) × A | 292.27 |  | ln(R) + ln(A) + ln(R) × ln(A) | 279.90 |  | R + ln(A) + R × ln(A) | 279.50 |

**Supplementary Table 4**. Results from the Egger's tests of publication bias on our findings. We used funnel tests of asymmetry with the sample size as the predictor. In all cases, we analyzed the response ratio across the entire dataset (denoted by the N variable label) followed by the significant covariate models that we present in the method (Eqn. 5, and Eqn. 6). *P*-values < 0.05 are in bold and indicate a significant publication bias.

| Attribute |  | *z* value |  | *p* value |
| --- | --- | --- | --- | --- |
| SOC content |  | 0.489 |  | 0.625 |
| SOC stock |  | 0.257 |  | 0.797 |
| Total nitrogen |  | 0.546 |  | 0.585 |
| Total phosphorus |  | 2.939 |  | **0.003** |
| Soil pH |  | -0.073 |  | 0.942 |
| Bulk density |  | -0.164 |  | 0.869 |
| Aboveground biomass |  | -0.177 |  | 0.859 |
| Belowground biomass |  | -5.082 |  | **<0.001** |

**Supplementary Table 5**. The effect (*P* values) of grazing exclusion duration (T), mean annual precipitation (P), and grassland type (L) on scaled natural log response ratios (ln*RR*) of soil organic carbon (SOC) content, SOC stock, aboveground biomass (AGB), belowground biomass (BGB), total nitrogen (TN), total phosphorus (TP), pH and bulk density (BD), DF, degrees of freedom; VIF, variance inflation factor. *P* values below 0.05 are significant and in bold.

| Source | Estimate |  | DF |  | F |  | *P* |  | VIF |
| --- | --- | --- | --- | --- | --- | --- | --- | --- | --- |
| **SOC content (R^2^_marginal_ = 0.087, R^2^_conditional_ = 0.409)** |  |  |  |  |  |  |  |  |  |
| scale(log(P)) | 0.085 |  | 1, 270.97 |  | 11.66 |  | **<0.001** |  | 1.27 |
| scale(log(T)) | 0.120 |  | 1, 764.50 |  | 76.45 |  | **<0.001** |  | 1.00 |
| scale(log(P)) × scale(log(T)) | 0.042 |  | 1, 761.15 |  | 7.61 |  | **0.005** |  | 1.27 |
| **SS stock (R^2^_marginal_ = 0.066, R^2^_conditional_ = 0.409)** |  |  |  |  |  |  |  |  |  |
| scale(log(P)) | 0.075 |  | 1, 269.71 |  | 9.41 |  | **0.002** |  | 1.26 |
| scale(log(T)) | 0.098 |  | 1, 765.48 |  | 54.77 |  | **<0.001** |  | 1.00 |
| scale(log(P)) × scale(log(T)) | 0.032 |  | 1, 763.25 |  | 4.94 |  | **0.026** |  | 1.26 |
| **AGB (R^2^_marginal_ = 0.124, R^2^_conditional_ = 0.731)** |  |  |  |  |  |  |  |  |  |
| scale(log(P)) | 0.125 |  | 1, 203.42 |  | 19.14 |  | **<0.001** |  | 2.77 |
| scale(log(T)) | -0.133 |  | 1, 181.13 |  | 40.88 |  | **<0.001** |  | 4.69 |
| scale(log(P)) × scale(log(T)) | 0.112 |  | 1, 176.97 |  | 7.09 |  | **<0.001** |  | 1.26 |
| L | / |  | 3, 78.06 |  | 0.66 |  | **0.008** |  | 1.46 |
| scale(log(P)) × L | / |  | 3, 176.10 |  | 9.44 |  | **<0.001** |  | 1.63 |
| scale(log(T)) × L | / |  | 3, 176.44 |  | 11.96 |  | **<0.001** |  | 1.85 |
| **BGB (R^2^_marginal_ = 0.103, R^2^_conditional_ = 0.652)** |  |  |  |  |  |  |  |  |  |
| scale(log(P)) | 0.008 |  | 1, 92.25 |  | 0.01 |  | 0.930 |  | 1.33 |
| scale(log(T)) | -0.108 |  | 1, 141.36 |  | 12.25 |  | **<0.001** |  | 3.83 |
| scale(log(P)) × scale(log(T)) | 0.166 |  | 3, 147.40 |  | 2.94 |  | 0.088 |  | 1.10 |
| L | / |  | 3, 52.05 |  | 0.32 |  | 0.810 |  | 1.80 |
| scale(log(T)) × L | / |  | 3, 132.62 |  | 11.61 |  | **<0.001** |  | 1.65 |
| **TN (R^2^_marginal_ = 0.036, R^2^_conditional_ = 0.315)** |  |  |  |  |  |  |  |  |  |
| scale(log(P)) | 0.021 |  | 1, 243.41 |  | 0.52 |  | 0.468 |  | 1.28 |
| scale(log(T)) | 0.096 |  | 1, 605.86 |  | 33.59 |  | **<0.001** |  | 1.00 |
| scale(log(P)) × scale(log(T)) | 0.010 |  | 1, 601.09 |  | 0.36 |  | 0.546 |  | 1.28 |
| **TP (R^2^_marginal_ = 0.057, R^2^_conditional_ = 0.534)** |  |  |  |  |  |  |  |  |  |
| scale(log(P)) | 0.083 |  | 1, 77.78 |  | 6.03 |  | **0.016** |  | 1.09 |
| scale(log(T)) | 0.050 |  | 1, 214.82 |  | 8.96 |  | **0.003** |  | 1.01 |
| scale(log(P)) × scale(log(T)) | 0.033 |  | 1, 209.057 |  | 3.59 |  | 0.059 |  | 1.10 |
| **pH (R^2^_marginal_ = 0.095, R^2^_conditional_ = 0.572)** |  |  |  |  |  |  |  |  |  |
| scale(log(P)) | -0.008 |  | 1, 124.30 |  | 3.11 |  | 0.079 |  | 1.12 |
| scale(log(T)) | -0.017 |  | 1, 220.47 |  | 33.18 |  | **<0.001** |  | 1.02 |
| scale(log(P)) × scale(log(T)) | -0.008 |  | 1, 205.61 |  | 8.95 |  | **0.003** |  | 1.13 |
| **BD (R^2^_marginal_ = 0.069, R^2^_conditional_ = 0.466)** |  |  |  |  |  |  |  |  |  |
| scale(log(P)) | -0.010 |  | 1, 267.31 |  | 5.04 |  | **0.025** |  | 1.22 |
| scale(log(T)) | -0.020 |  | 1, 764.26 |  | 74.51 |  | **<0.001** |  | 1.00 |
| scale(log(P)) × scale(log(T)) | -0.008 |  | 1, 765.69 |  | 9.32 |  | **0.002** |  | 1.23 |


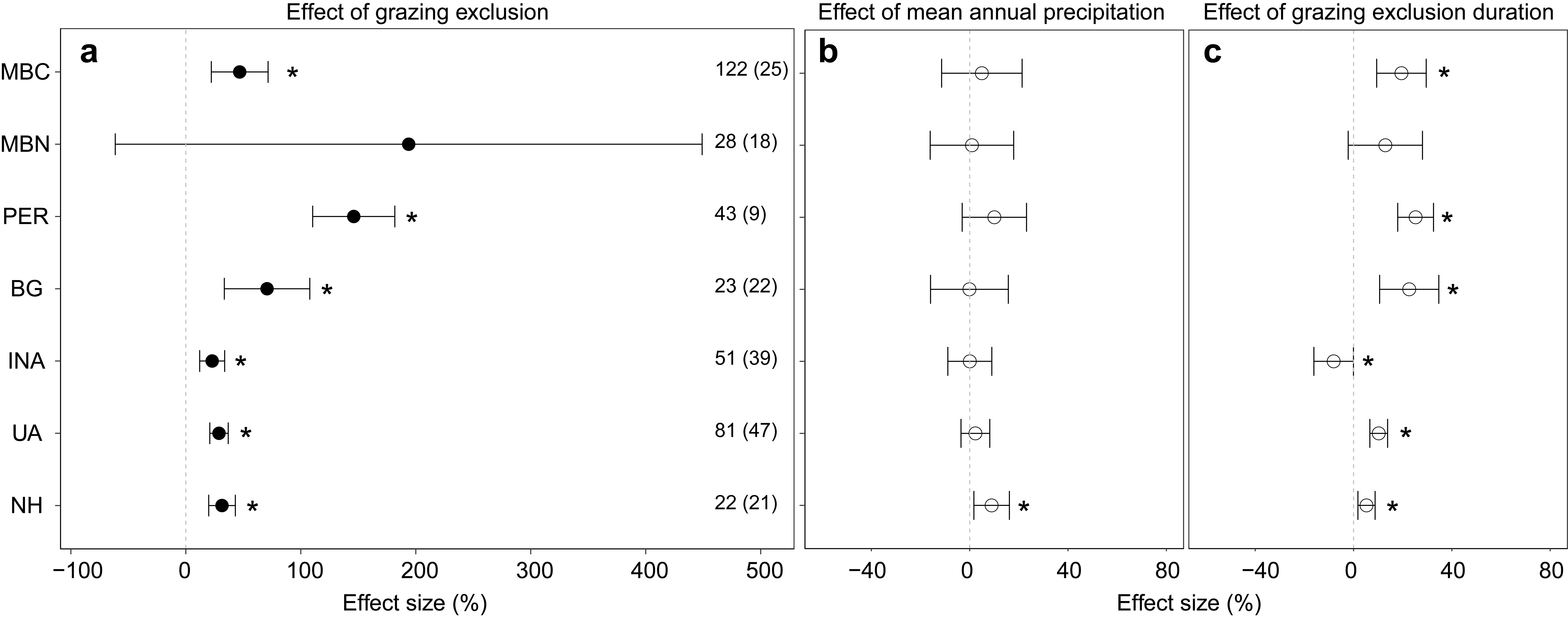


**Supplementary Figure 6**. The effects of grazing exclusion on environmental attributes in relation to mean annual precipitation and grazing exclusion duration. **a** The grazing exclusion. **b** The mean annual precipitation. **c** Grazing exclusion duration (years). The effects represent the estimated coefficients of the mean annual precipitation and grazing exclusion duration. Values (estimated *β_1_* and *β_2_* in Equation (3), respectively, see Methods) are mean ± 95% confidence intervals. MBC: microbial biomass carbon, MBN: microbial biomass nitrogen, PER: peroxidase, BG: β-1,4-glucosidase, INA: Invertase, UA: Urease, NH: Protease. The number of observations in **a** is shown without parentheses above each attribute together with the number of studies in parentheses. Source data are provided as a Source Data file.


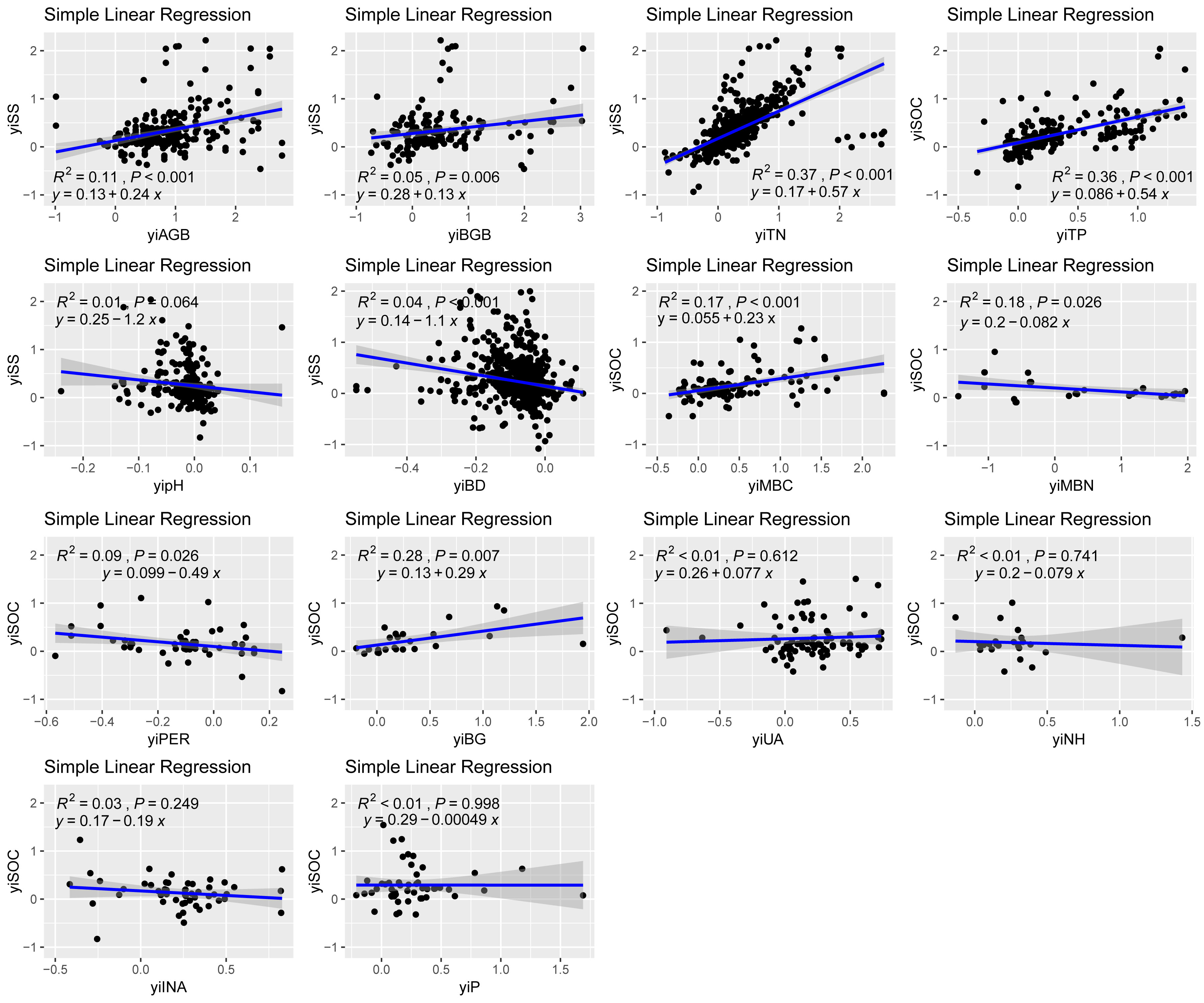


**Supplementary Figure 7**. Linear regression analysis between different environmental attributes and soil organic carbon in grasslands. SS: soil organic carbon stock, SOC: soil organic carbon, AGB: aboveground biomass, BGB: belowground biomass, TN: total nitrogen, TP: total phosphorus, pH: soil pH, BD: soil bulk density, MBC: microbial biomass carbon, MBN: microbial biomass nitrogen, PER: Peroxidase, BG: β-1,4-glucosidase, UA: Urease, NH: Proteases, INA: Invertase, P: Phosphatase.

**Supplementary Table 6.** Global spatial autocorrelation coefficients (Moran’s *I* value) based on different spatial weights for natural log response ratios (ln*RRs*) of soil organic carbon (SOC) content and stock. *P*-values < 0.05 indicate spatial autocorrelation existed.

|  | Spatial autocorrelation | | |
| --- | --- | --- | --- |
| Index | Moran’s *I* value |  | *P* |
| SOC content | -1.201 |  | 0.885 |
| SOC stock | -0.821 |  | 0.794 |


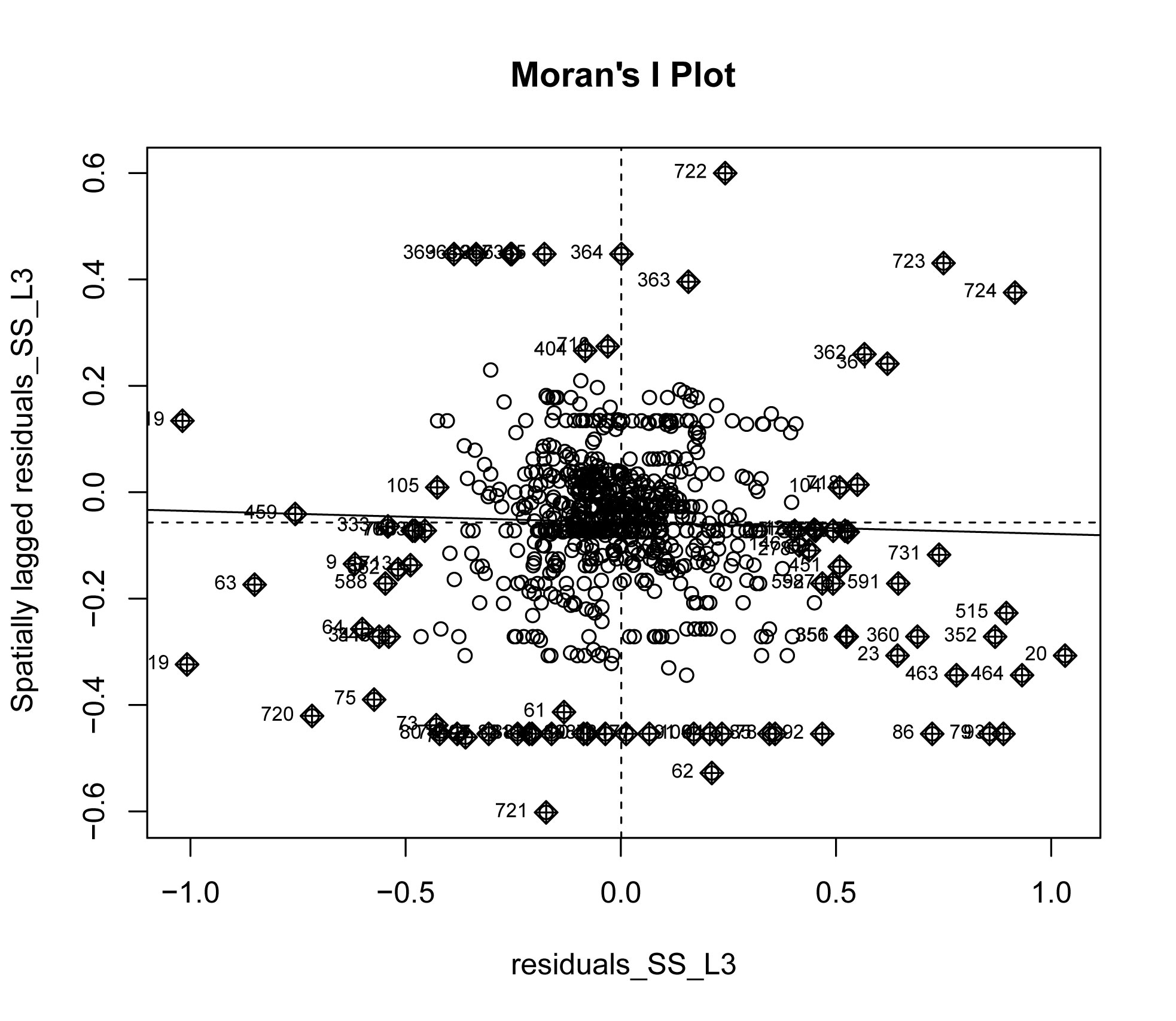


**Supplementary Figure 8**. Linear regression analysis between different environmental attributes and soil organic carbon in grasslands


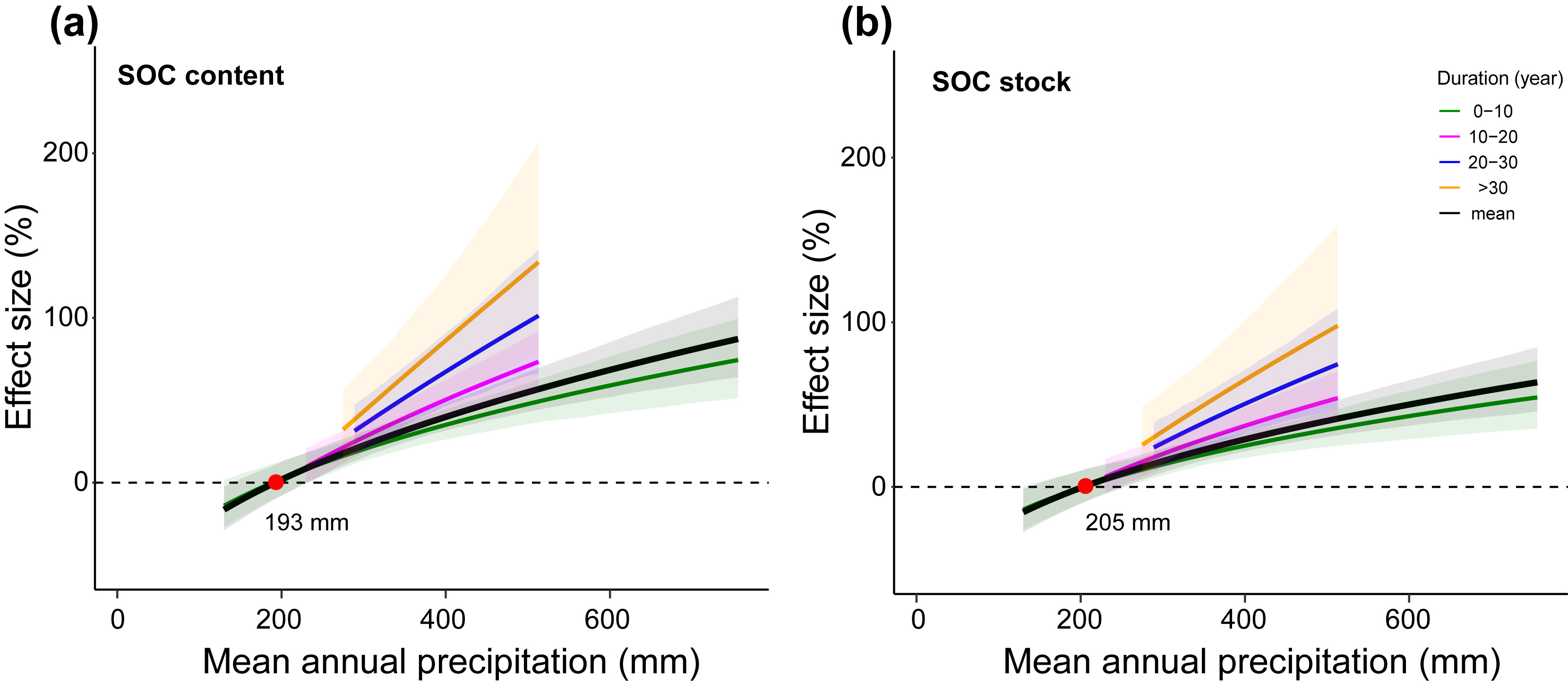


**Supplementary Figure 9**. The annual average precipitation threshold for soil carbon sequestration under different grazing exclusion duration, based on the effect size for SOC content (a) and SOC stock (b)

**Supplementary Table 7.** AICc-based model-selection summary for testing additional MAP×MAT and MAP×pH moderation of grazing-exclusion effects on soil organic carbon (SOC).

| Response | Tested moderator | Best AICs model  (including terms) | AICs | Weight | Best model including MAP×moderator | ΔAIC | Weight | Interpretation |
| --- | --- | --- | --- | --- | --- | --- | --- | --- |
| SOC content | MAP×MAT | (MAP, Duration, MAP×Duration) | 135.5 | 0.795 | model with MAP×MAT | 10.13 | 0.005 | MAP×MAT not supported |
| SOC stock | MAP×MAT | (MAP, Duration, MAP×Duration) | 129.4 | 0.460 | model with MAP×MAT | 8.87 | 0.005 | MAP×MAT not supported |
| SOC content | MAP×pH | (MAP, Duration, MAP×Duration) | 135.5 | 0.948 | model with MAP×pH | 11.55 | 0.003 | MAP×pH not supported |
| SOC stock | MAP×pH | (MAP, Duration, MAP×Duration) | 129.4 | 0.678 | model with MAP×pH | 12.30 | 0.001 | MAP×pHnot supported |

Note: Mixed-effects meta-regression model selection comparing the AICc-best model with the best candidate model that includes MAP×MAT or MAP×pH. ΔAICc is relative to the AICc-best model and w denotes Akaike weight; MAP = mean annual precipitation, MAT = mean annual temperature, and pH refers to baseline soil pH in grazed controls (pH.ck) when used as a moderator. Models with ΔAICc > 10 and near-zero w were treated as unsupported.

**Supplementary Methods**

**The interpretation of the effects of induced or reduced mean annual precipitation on soil organic carbon stock over time.**

The ln*RR* when the mean annual precipitation was *R_1_* for a period of *T*:

$\ln RR_{1}=\beta_{0}+\beta_{1}\cdot\ln(R_{1})+\beta_{2}\cdot T+\beta_{3}\cdot\ln(R_{1})\times T+\pi_{study}+\varepsilon$ (1)

The ln*RR* when mean annual precipitation was *R_α_* (α % lower of higher mean annual precipitation than *R_1_*) for a duration of *T*:

$\ln RR_{\alpha}=\beta_{0}+\beta_{1}\cdot\ln(R_{\alpha})+\beta_{2}\cdot T+\beta_{3}\cdot\ln(R_{\alpha})\times T+\pi_{study}+\varepsilon$ (2)

Supplementary Equation 2 - Supplementary Equation 1:

$\ln RR_{\alpha}-\ln RR_{1}=(\beta_{1}+\beta_{3}\cdot A)\times(\ln(R_{\alpha})-\ln(R_{1}))$ (3)

$\ln(RR_{\alpha}/RR_{1})=\ln((R_{\alpha}/R_{1})^{\beta_{1}+\beta_{3}\cdot A})$ (4)

$(X_{t_{-}\alpha}/X_{c_{-}\alpha})/(X_{t_{-}1}/X_{c_{-}1})=RR_{\alpha}/RR_{1}=(R_{\alpha}/R_{1})^{\beta_{1}+\beta_{3}\cdot A}$ (5)

We assumed that the mean value of duration (*X_c_*) did not vary with the different mean annual precipitation, i.e., *X_c_α_* was not different from *X_c_1_*.
